# Single-cell RNA-sequencing reveals radiochemotherapy-induced innate immune activation and MHC-II upregulation in cervical cancer

**DOI:** 10.1038/s41392-022-01264-9

**Published:** 2023-01-30

**Authors:** Chao Liu, Xiaohui Li, Qingyu Huang, Min Zhang, Tianyu Lei, Fuhao Wang, Wenxue Zou, Rui Huang, Xiaoyu Hu, Cong Wang, Xiaoling Zhang, Bing Sun, Ligang Xing, Jinbo Yue, Jinming Yu

**Affiliations:** 1 Department of Radiation Oncology and Shandong Provincial Key Laboratory of Radiation Oncology, Shandong Cancer Hospital and Institute, Shandong First Medical University and Shandong Academy of Medical Sciences, Jinan, 250117 China; 2 Research Unit of Radiation Oncology, Chinese Academy of Medical Sciences, Jinan, 250117 China; 3 Department of Nephrology, First Medical Center of Chinese PLA General Hospital, Beijing, 100853 China; 4 Department of Oncology, Renmin Hospital of Wuhan University, Wuhan, 430060 China; 5 School of Clinical Medicine, Weifang Medical University, Weifang, 261053 China; 6 Department of Gynecologic Oncology, Shandong Cancer Hospital and Institute, Shandong First Medical University and Shandong Academy of Medical Sciences, Jinan, 250117 China; 7 Department of Radiation Oncology, Fifth Medical Center of Chinese PLA General Hospital, Beijing, 100071 China

**Keywords:** Cancer therapy, Gynaecological cancer

## Abstract

Radiochemotherapy (RCT) is a powerful treatment for cervical cancer, which affects not only malignant cells but also the immune and stromal compartments of the tumor. Understanding the remodeling of the local ecosystem induced by RCT would provide valuable insights into improving treatment strategies for cervical cancer. In this study, we applied single-cell RNA-sequencing to paired pre- and post-RCT tumor biopsies from patients with cervical cancer and adjacent normal cervical tissues. We found that the residual population of epithelial cells post-RCT showed upregulated expression of MHC class II genes. Moreover, RCT led to the accumulation of monocytic myeloid-derived suppressor cells with increased pro-inflammatory features and CD16^+^ NK cells with a higher cytotoxic gene expression signature. However, subclusters of T cells showed no significant increase in the expression of cytotoxic features post-RCT. These results reveal the complex responses of the tumor ecosystem to RCT, providing evidence of activation of innate immunity and MHC-II upregulation in cervical cancer.

## Introduction

Cervical cancer (CC) is one of the most prevalent gynecological tumors and the fourth leading cause of female cancer-related mortality, with ~604,127 new cases of CC and 341,831 CC-related deaths reported worldwide.^1^ Pelvic radiotherapy with concurrent chemotherapy has become the standard treatment for locally advanced cervical cancer, with randomized clinical trials showing a typical response rate of ~90% and an average 5-year survival rate of 72%.^2,3^ However, ~20% of patients with CC experience pelvic recurrence and/or distant metastasis in the five years following radiochemotherapy (RCT), which often leads to death.^4,5^ Understanding the response of epithelial/tumor cells and tumor microenvironment (TME) to RCT would provide valuable insight into the processes underlying a successful response to CC treatment.

It is well-documented that RCT suppresses tumor growth and modulates anti-tumor immunity in various cancers.^6,7^ In patients with CC, studies of bulk RNA-sequencing of immune cells have shown that RCT stimulates the expression of the co-stimulatory molecule CD28^8^ and downregulates the expression of inhibitory molecules PD-1/PD-L1.^9^ At the same time, multispectral flow cytometry of cervical smears revealed an early decrease in the number of T cells in the RCT-treated cervical TME, followed by an increase in their number at a later stage. However, although these findings are important, bulk analyses of pre-selected cell types provide a view of the averaged global gene expression patterns from the cell population within the tissue type but do not differentiate the cell types in the sample. Single-cell RNA-sequencing (scRNA-seq) has revolutionized our ability to comprehensively analyze gene expression patterns across a range of cell types in a tissue sample; it has recently been used to generate detailed cellular landscapes of the TME in various types of solid tumors.^10,11^ However, this powerful technique has yet to be applied to elucidate the effect of RCT on epithelial cells and TME in patients with CC.

In this study, we performed scRNA-seq on 10 paired biopsies of CC tumors collected pre- and post-RCT and three samples of adjacent normal cervical tissues to characterize the CC ecosystem and identify dynamic changes in the tumor and its microenvironment. We uncovered the presence of a heterogenous population of epithelial cells remaining after RCT and revealed the activation of innate immune cells, but not T cells, during treatment. Taken together, these data show the modulating effect of RCT on the cervical ecosystem in patients with CC at a single-cell resolution.

## Results

### Single-cell transcriptome identifies CC ecosystem dynamics during RCT

**Fig. 1:**
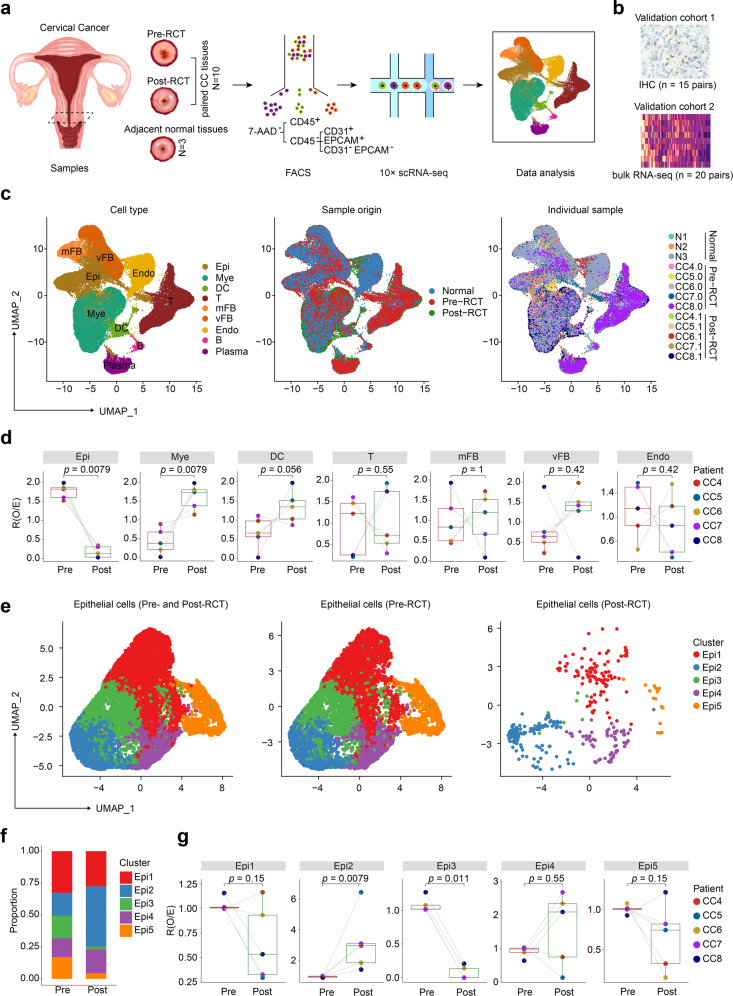
Single-cell landscape of cervical cancer pre- and post-RCT. **a** Schematic overview of the strategy of scRNA-seq in this study. **b** Validation cohort 1 (15 pairs of pre-RCT and post-RCT CC samples for IHC) and cohort 2 (20 pairs of pre-RCT and post-RCT bulk RNA-seq data). **c** Uniform manifold approximation and projection (UMAP) visualization of all identified cell clusters. Colors represent cell types (left panel), sample origin (middle panel), and individual samples (right panel). **d** Box plots showing the relative fold change in the abundance of each cell cluster compared between pre- and post-RCT samples. The colors of dots and squares represent individual patients and sample groups, respectively. The *p* values correspond to the paired Wilcoxon tests. **e** UMAP visualization of the 5 Epi subclusters in all (left panel), pre-RCT (middle panel), and post-RCT samples (right panel). **f** Bar plots showing the relative proportions of the 5 Epi subclusters in all epithelial cells in pre/post-RCT samples. **g** Box plots showing the relative fold change in the abundance of each Epi cell cluster compared between pre- and post-RCT samples. The colors of dots and squares represent individual patients and sample groups, respectively. The *p* values correspond to the paired Wilcoxon tests

We collected 10 paired samples of tumor tissue from five patients with CC at two distinct time points: 1 week before RCT (pre-RCT) and 3 weeks later, during their ongoing treatment (post-RCT). Alongside, we collected three samples of adjacent normal cervical tissue from 2 patients with cervical intraepithelial neoplasia for scRNA-seq (Fig. 1a). In addition, we aimed to perform validated testing on 2 validation cohorts: validation cohort 1, comprising 15 pairs of pre-RCT and post-RCT CC samples employed for immunohistochemistry (IHC), and validation cohort 2, comprising 20 pairs of bulk RNA-seq data of pre-RCT and post-RCT CC samples (Fig. 1b).

For scRNA-seq, we enriched the samples for immune (CD45^+^), epithelial (CD45^−^ EPCAM^+^), endothelial (CD45^−^ CD31^+^), and fibroblastic cells (CD45^−^ EpCAM^−^ CD31^−^), and subjected them to droplet-based scRNA-seq. After quality control, we analyzed the data from 60,550 single cells, with an average of 2730 expressed genes per cell detected (Fig. 1c and Supplementary Fig. 1a, b). Based on the differential expression of prototypical signature genes in the cells (Supplementary Fig. 1c, d), we manually annotated nine cell clusters: myeloid cells (“Mye”, expressing *CD14* and *CD68*), T cells (“T”, expressing *CD3D* and *CD3E*), plasma cells (“Plasma”, expressing *SDC1* and *SLAMF7*), B cells (“B”, expressing *MS4A1* and *CD79B*), dendritic cells (“DC”, expressing *CD1C*), endothelial cells (“Endo”, expressing *CDH5* and *VWF*), epithelial cells (“Epi”, expressing *EPCAM*), vascular fibroblasts (“vFB”, expressing *RGS5* and *MYH11*), and matrix fibroblasts (“mFB”, expressing *LUM* and *DCN*). All 9 clusters were present in normal, pre-, and post-RCT cervical samples (Fig. 1c).

We next employed a pairwise analysis to examine the changes in the cellular composition of CC after RCT (Fig. 1d). As expected, the relative frequency of “Epi” was significantly reduced in all patients (*p* = 0.0079), consistent with treatment-induced necrosis and apoptosis of cancer cells. Moreover, the proportion of “Mye” was significantly elevated after RCT in all patients (*p* = 0.0079), indicating the recruitment of myeloid cells and their infiltration into the tumors. However, the relative changes in the abundance of the other 7 clusters varied widely between patients, highlighting heterogenous responses to treatment at the cellular level.

### Diverse RCT sensitivity and immunogenicity exist among epithelial cell subtypes

Next, we extracted data from epithelial cells for further sub-clustering, which yielded five subclusters that we termed Epi1-5 (Fig. 1e). Heatmap visualization of the top differentially expressed genes (DEGs) in each subcluster revealed the genes associated with tumorigenesis and tumor escape from immune surveillance.^12,13^ Mesenchyme-associated genes, including *IGFBP5, TAGLN, COL1A1*, and *ACTA2*, were most abundantly expressed in Epi1; immune-related genes, including *LCN2* and *DEFB1*, were most abundantly expressed in Epi2; and cell cycle genes, including *TOP2A* and *MKI67*, were most abundantly expressed in Epi5 (Supplementary Fig. 2a). We validated these findings using the Gene Set Variation Analysis (GSVA), which confirmed that: (a) WNT/β-Catenin and epithelial-mesenchymal-transition characterized Epi1; (b) complement and inflammatory signaling were hallmarks of Epi2; and (c) DNA repair and a high S/G2M ratio was typically seen in Epi5 (Supplementary Fig. 2b). Interestingly, we found that RCT altered the relative proportions of the five subclusters, seen most evidently as an increase in the proportion of Epi2 and a decrease in the proportion of Epi3 (Fig. 1f, g). In conclusion, these data demonstrated that the subsets of epithelial cells in CC displayed heterogeneous proliferation, tumor immunology, and sensitivity to RCT.

**Fig. 2:**
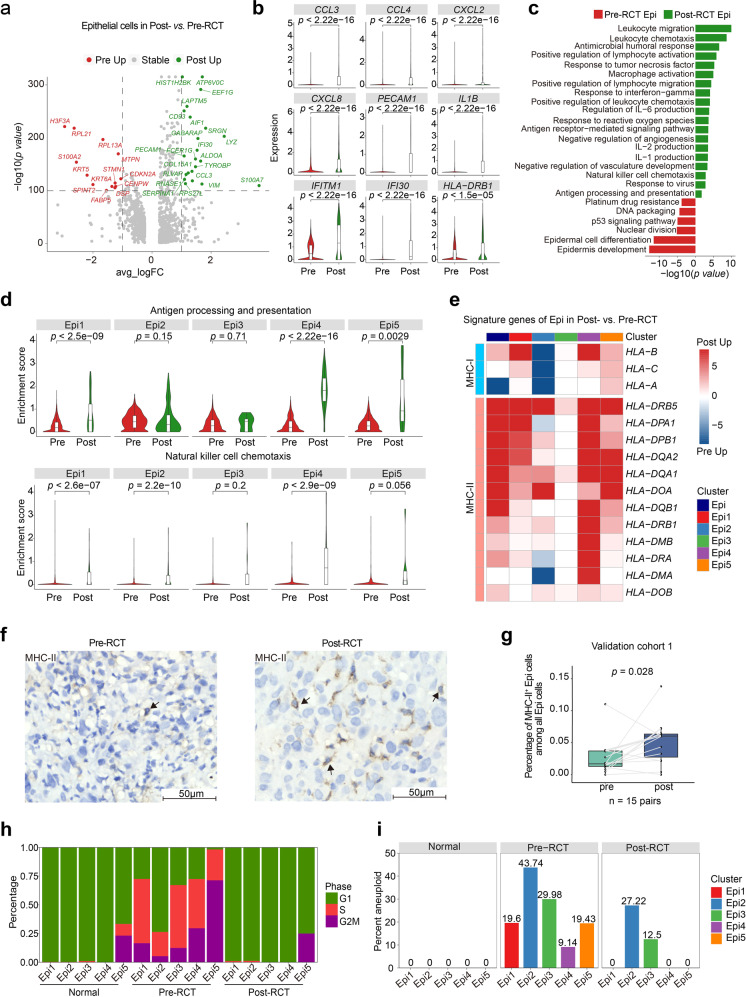
RCT activates the expression of immune response genes in epithelial cells. **a** Volcano plot showing the changes in gene expression in epithelial cells pre- and post-RCT. The colored dots represent the top most variable genes. **b** Violin plots showing the expression level of the indicated genes in epithelial cells pre- and post-RCT (two-sided Wilcoxon test). **c** The enriched GO terms for epithelial cells in pre- versus post-RCT samples. **d** Violin plots showing the indicated pathway enrichment of each Epi subcluster pre- and post-RCT (two-sided Wilcoxon test). **e** Heatmap showing the relative changes in MHC class I and II gene expression in all epithelial cells and each Epi subcluster in pre- and post-RCT groups. The intensity of the color indicates the extent of upregulation post-RCT (red) or pre-RCT (blue). **f** Representative images of IHC staining of MHC-II (HLA-DR + DP + DQ) in epithelial cells in paired pre-RCT and post-RCT FFPE tissues. Scale bar, 50 μm. **g** Box plot showing the fraction of MHC-II positive Epi cells among all Epi cells in pre-RCT and post-RCT samples based on IHC results in **f**. The *p* value corresponds to the paired *t* test. **h** Bar plots showing the proportion of cells in each cell cycle phase within each Epi subcluster normal, pre-RCT, and post-RCT samples. **i** Bar plots showing the percentage of aneuploid cells (tumor cells) within each Epi subcluster in normal, pre-RCT, and post-RCT samples

We then examined how RCT affected immune response-related features of epithelial cell subclusters. Immune-associated DEG and gene ontology (GO) term analysis provided evidence of overall immunogenicity-enhancing effects of RCT; these included increased expression of genes involved in leukocyte chemotaxis, lymphocyte activation, and antigen presentation and processing (Fig. 2a–c and Supplementary Fig. 3a, b). Specifically, the genes involved in antigen presentation and processing, including MHC-II, were significantly upregulated in overall epithelial cells and Epi1/4/5 post-RCT, while the pathways of antigen processing and presentation remained relatively stable in Epi2/3, with only Epi2 showing some downregulation of MHC-I expression (Fig. 2d, e). Similar changes in the expression of signature genes were seen in patients from our sample: in most patients, the observed gene expression patterns corresponded to overall gene expression changes, although one patient had a different gene expression pattern, likely attributed to the cancer patient heterogeneity (Supplementary Fig. 3c). IHC staining of samples from validation cohort 1 confirmed the upregulation of MHC-II genes in epithelial cells post-RCT (Fig. 2f, g). Taken together, we demonstrated that RCT induced a broad immune-related response in epithelial cells.

Subsequently, we sought to assess how RCT affected the malignant features of epithelial cell subsets. We found that RCT downregulated the expression of genes associated with malignant CC features, including *KRT6A*, *KRT15*, *KRT16*, *KRT17*, *CDKN2A*, and *ERBB2* (Supplementary Fig. 3d, e). Regarding the cell cycle, we observed a higher ratio of G2/S phase gene expression in pre-RCT samples compared to normal and post-RCT samples (Fig. 2h). Then, we evaluated the change in the number of tumor cells induced by RCT in each epithelial cell subcluster and observed an overall reduction. Notably, while the proportion of aneuploid cells (tumor cells) decreased to zero post-RCT in 3 of the 5 clusters, we detected a significant number of aneuploid cells remaining in Epi2/3 at 3 weeks post-RCT (Fig. 2i). Although the residual Epi2/3 clusters were not proliferating at that time point (Fig. 2h), Epi2 expressed relatively high levels of the SPRR and S100A family genes, which are precancerous and pro-metastatic markers (Supplementary Fig. 3d).^14–16^

Taken together, the above analyses showed that RCT induced an increase in the expression of genes associated with the immune response, notably MHC-II, in epithelial cells. However, at the same time, there remained a residual epithelial cell subset that was characterized by potential malignant features and low expression of genes involved in antigen presentation.

### RCT induces pro-inflammatory gene expression in myeloid cells

**Fig. 3:**
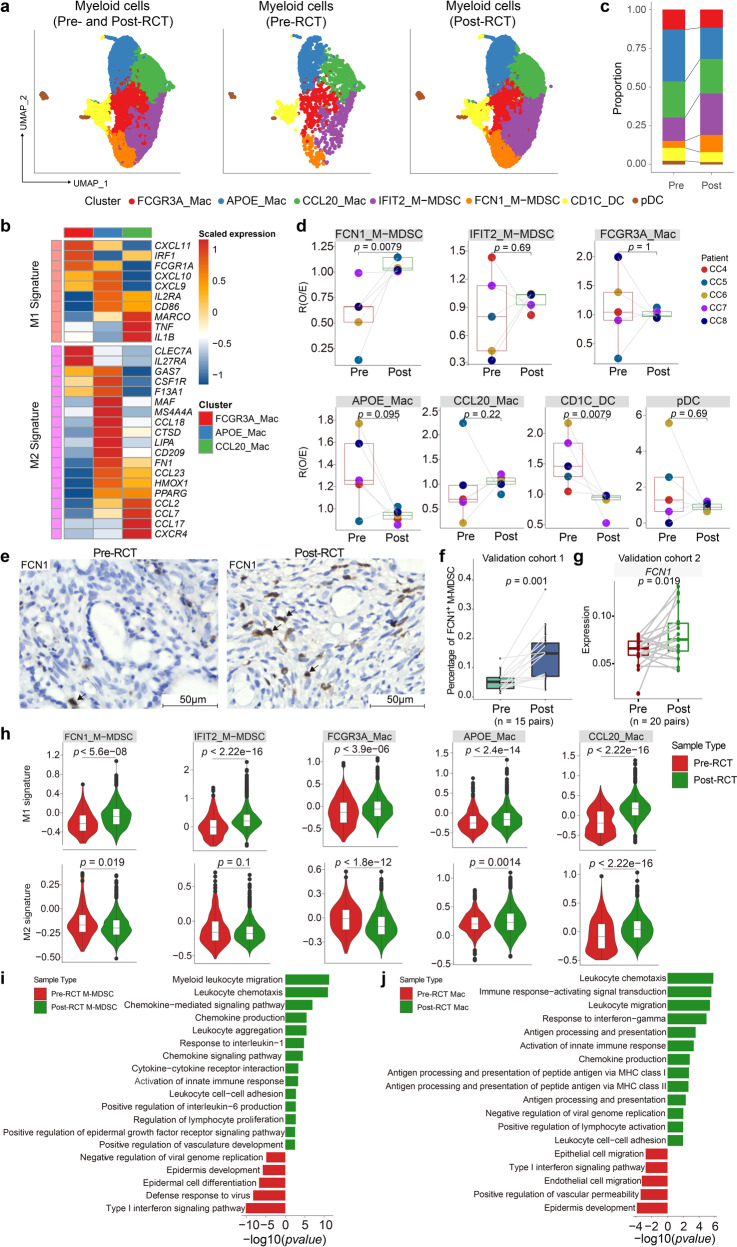
RCT increases FCN1_M-MDSC cells with elevated pro-inflammatory features. **a** UMAP visualization of the seven myeloid subclusters in all (left panel), pre-RCT (middle panel), and post-RCT (right panel) samples. **b** Heatmap showing the expression level of M1 and M2 signature genes in macrophage subclusters. **c** Bar plot showing the relative proportion of each myeloid cell subcluster in all myeloid cells in pre/post-RCT samples. **d** Box plots showing the fold change in the abundance of each myeloid cell subcluster compared between pre- and post-RCT samples. The colors of dots and squares represent patients and sample groups, respectively. The *p* values correspond to the paired Wilcoxon tests. **e** Representative images of IHC staining of FCN1 in paired pre-RCT and post-RCT FFPE tissues. Scale bar, 50 μm. **f** Box plot showing the fraction of FCN1^+^ M-MDSC cells in paired pre-RCT and post-RCT samples based on IHC results in **e**. The *p* value corresponds to the paired *t* test. **g** Box plot showing gene expression of *FCN1* in pre-RCT and post-RCT bulk RNA-seq data. The *p* value corresponds to the paired *t* test. **h** Violin plots showing the expression of M1 and M2 signature genesets in myeloid cell subclusters in pre- and post-RCT groups (two-sided Wilcoxon test). **i** GO term analysis of M-MDSC cells in pre-RCT versus post-RCT samples. **j** GO term analysis of macrophages in pre-RCT versus post-RCT samples

We next focused on myeloid cells, which influence tumor plasticity and progression.^17^ Based on the expression of marker genes, we categorized the myeloid cell population into seven heterogeneous subclusters: three subtypes of macrophages, two types of monocytic myeloid-derived suppressor cells (M-MDSCs), and two populations of DCs (Fig. 3a and Supplementary Fig. 4a, b). Two cell subclusters showed a notably pro-tumoral potential: APOE_Mac highly expressed the *C1Qs* complement gene, implying its role in efferocytosis, which may promote immunosuppressive TME;^18^ whereas CCL20_Mac expressed genes encoding immunosuppressive chemokines, such as *CCL20* and *CXCL8*.^19,20^ In contrast, cells within the IFIT2_M-MDSC cluster predominantly expressed pro-inflammatory genes, such as *IFIT2* and *CXCL10*, which may promote anti-tumor immunity. These data suggest that the myeloid cell population potentially contains both pro-tumor and anti-tumor subtypes. To further examine this phenomenon, we quantified pro-inflammatory M1- and anti-inflammatory M2-associated gene expression in macrophage subclusters and found that they exhibited a mixed M1 and M2 signature (Fig. 3b).

The overall composition of the myeloid cell compartment was also somewhat modified following RCT (Fig. 3c, d): most notably, there was a significant increase in the relative proportion of FCN1_M-MDSC and a decrease in the relative proportion of CD1C_DC. The enrichment of FCN1_M-MDSC was validated by IHC performed on the samples from the validation cohort 1 (Fig. 3e, f) and the pre- vs. post-RCT comparison of the bulk RNA-seq data from the validation cohort 2 (Fig. 3g). Regardless of the proportion of each cluster, we found that pro-inflammatory signatures were elevated in all clusters post-RCT (Fig. 3h). By investigating gene expression changes in the myeloid cells, we found that RCT significantly upregulated the expression of genes involved in chemotaxis and antigen processing and presentation (Supplementary Fig. 5a–f). Accordingly, the GO analysis revealed that the expression of genes involved in leukocyte migration and activation was increased in M-MDSCs, macrophages, and DCs post-RCT (Fig. 3i, j, and Supplementary Fig. 5g).

In addition, the enrichment of antigen processing and presentation was demonstrated in M-MDSCs and DCs post-RCT (Supplementary Fig. 5h, i). In accordance with previous studies that have shown that irradiation recruits MDSCs,^21,22^ we observed an enriched term of chemotaxis and migration in M-MDSCs (Fig. 3i), which might have contributed to an increase in FCN1_M-MDSC (Fig. 3d). Furthermore, pro- and anti-inflammatory features of the FCN1_M-MDSC subcluster were significantly upregulated and downregulated, respectively (Fig. 3h). Previous reports showed that polymorphonuclear MDSCs represented the main subset recruited in a mouse model of lung and bladder cancer.^23,24^ In our study, MDSCs that migrated toward CC microenvironment were monocytic, and granulocytes were not detected. In summary, RCT altered the immunological balance in CC tumors by inducing pro-inflammatory gene expression in myeloid cells and promoting the accumulation of FCN1_M-MDSCs with upregulated pro-inflammatory and downregulated anti-inflammatory features.

### The lymphocyte compartment is profoundly affected by RCT

**Fig. 4:**
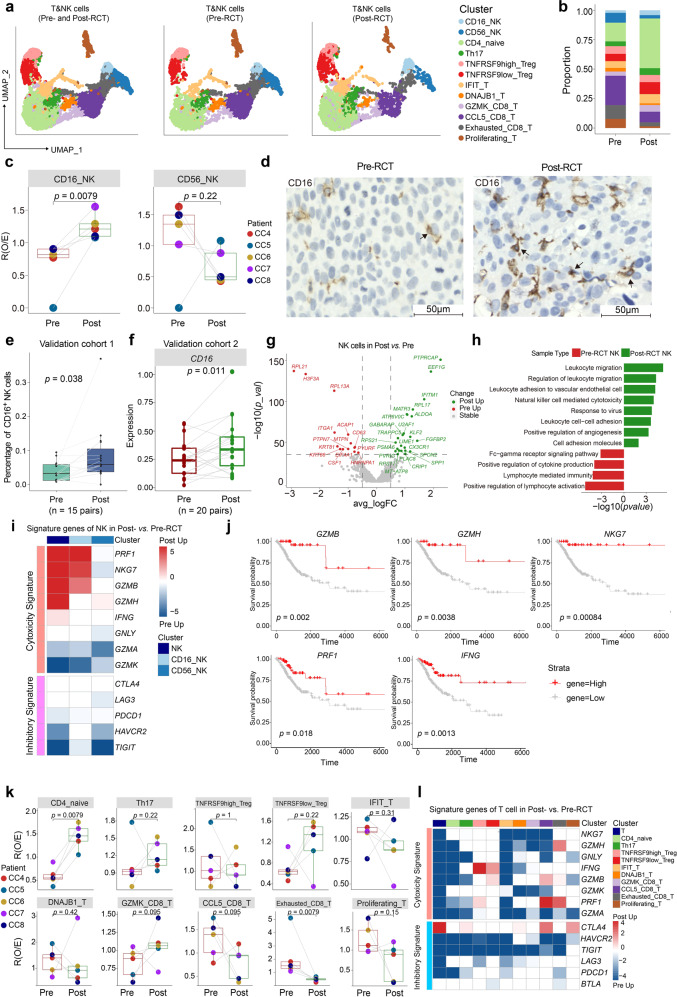
RCT increases the abundance of CD16_NK cells with cytotoxic features, while T-cell subclusters show decreased cytotoxic features. **a** UMAP visualization of 12 lymphocyte subclusters in all (left panel), pre-RCT (middle panel), and post-RCT (right panel) samples. **b** Bar plot showing the relative abundance of 12 subclusters in all lymphocytes in pre/post-RCT samples. **c** Box plot showing the fold change in the abundance of CD16_NK and CD56_NK cells post-RCT. The *p* values correspond to the paired Wilcoxon tests. **d** Representative images of IHC staining of CD16 in paired pre-RCT and post-RCT FFPE tissues. Scale bar, 50 μm. **e** Box plot showing the fraction of CD16^+^ NK cells in paired pre-RCT and post-RCT samples based on IHC results in **d**. The *p* value corresponds to the paired *t* test. **f** Box plot showing gene expression of CD16 in pre-RCT and post-RCT bulk RNA-seq data. The *p* value corresponds to the paired *t* test. **g** Volcano plots showing the gene expression changes in NK cells pre- and post-RCT. The colored dots represent the top most variable genes. **h** GO term analysis of NK cells in pre-RCT versus post-RCT samples. **i** Heatmap showing the relative changes in cytotoxic and inhibitory signature gene expression in all NK cells and individual NK cell subclusters between pre- and post-RCT groups. The intensity of the color indicates the extent of upregulation in post-RCT (red) or pre-RCT (blue) samples. **j** Kaplan-Meier analysis of overall survival in 295 TCGA bulk RNA-seq data of patients with CC, separated by high and low expression of cytotoxic genes. The *p* values correspond to the log-rank tests. **k** Bar plots showing the fold change in the abundance of 10 T cell subclusters in pre/post-RCT samples. The colors of dots and squares represent patients and sample groups, respectively. The *p* values correspond to the paired Wilcoxon tests. **l** Heatmap showing the changes in the expression level of cytotoxic and inhibitory signature genes in all T cells and T cell subclusters between pre- and post-RCT groups. The intensity of the color indicates the extent of upregulation in post-RCT (red) or pre-RCT (blue) samples

Among lymphocytes, NK and T cells are most renowned for their potential pro- or anti-tumor effects. In our data, we detected 10 subclusters of T cells along with 2 subclusters of NK cells (Fig. 4a and Supplementary Fig. 6a). It was revealed that the expression of genes encoding CD4 and those involved in the regulation of lymphocytogenesis, including *IL7R* and *CCR7*, was enriched in the CD4_Naive cluster. At the same time, CD16_NK and GZMK_CD8 expressed a clear cytotoxic signature, whereas Exhausted_CD8 showed high expression of the *LAG3* and *HAVCR2* exhaustion marker genes (Supplementary Fig. 6b–d).

Subsequently, we studied changes in the relative abundance and expression of genes in these clusters following RCT. The proportion of CD16_NK cells was significantly increased, while the proportion of CD56_NK cells remained unchanged (Fig. 4b, c). The increase of CD16_NK cells was validated by performing IHC staining of samples from validation cohort 1 (Fig. 4d, e) and examining changes in CD16 expression from validation cohort 2 (Fig. 4f). Considering DEG and GO term analyses of NK cells pre- and post-RCT, we found increased expression of genes associated with leukocyte migration in both NK subpopulations post-RCT, and genes involved in cytotoxicity in the CD16_NK cell subpopulation (Fig. 4g, h, and Supplementary Fig. 7a, b). In support of the cytotoxic role of CD16_NK cells and in agreement with the results of previous studies showing that CD16_NK cells mediate antibody-dependent cellular cytotoxicity without cytokine stimulation,^25^ CD16_NK cells showed increased expression of *GZMB, GZMH*, and *NKG7* post-RCT; protein products of these genes are expressed in cytotoxic granules of NK cells (Fig. 4i).^26,27^ The samples of most patients had increased expression of these cytotoxic genes, with the exception of one patient who had heterogeneity in the gene expression pattern (Supplementary Fig. 7c). We then evaluated the relationship between the expression of these upregulated cytotoxic genes and the survival of patients with CC using bulk expression data. We found that the expression of the cytotoxic genes positively correlated with patient survival (Fig. 4j).

Along with these changes in the NK cell compartment, an even more pronounced restructuring occurred in the local T cell population after RCT. Most notably, the proportion of CD4_naive cells showed a significant increase, whereas the proportion of Exhausted_CD8_T cells showed a significant decrease (Fig. 4k). Although the inhibitory features were downregulated across all subclusters of T cells, we did not find a significant increase in cytotoxic score, unlike what was observed in NK cells (Fig. 4l). Although the immune function of naïve T cells is not as strong as that of mature effector lymphocytes, naïve T cells can affect immunity to cancer by replenishing T cells in the TME. Given the abundance of other CD4^+^ T cell clusters, which were comparable before and after RCT, the differentiation of CD4_naive cells may have been impaired. Moreover, additional sampling points may be required to properly study the dynamics of other T cell populations during RCT. Taken together, we showed that CD16^+^ NK cells were recruited into CC microenvironment during RCT. These cells expressed increased levels of genes encoding cytotoxic molecules that were associated with favorable survival. In contrast, T cell subclusters showed no significant increase in cytotoxic properties during RCT treatment.

### Cancer-associated fibroblasts exhibit reduced tumor-promoting features during RCT

**Fig. 5:**
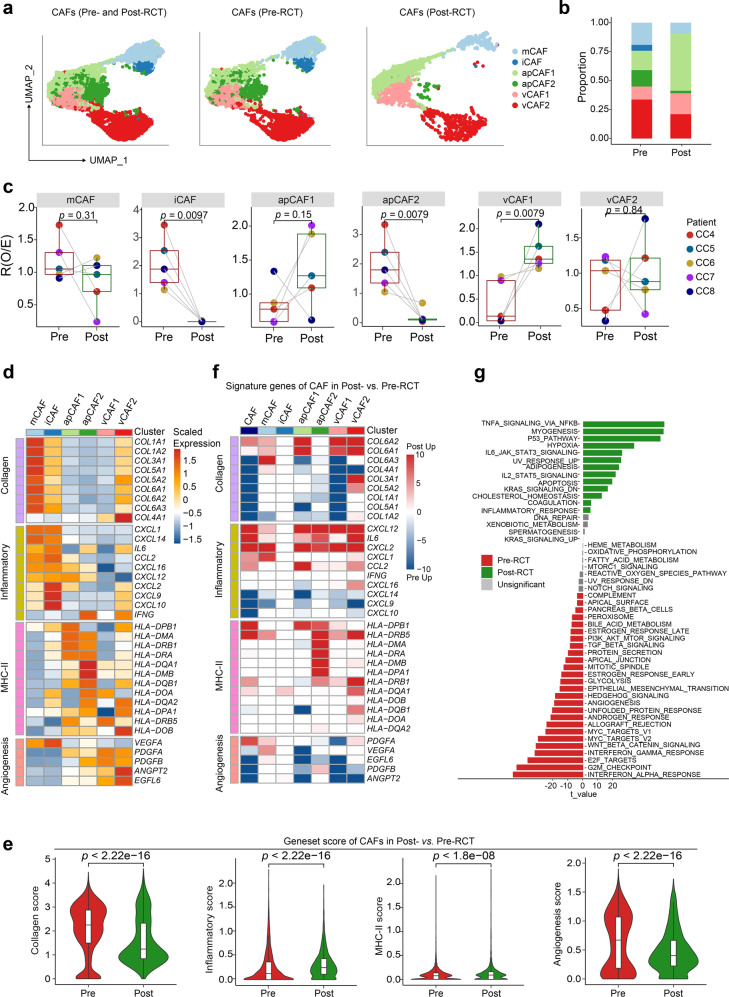
RCT weakens the tumor-promoting features of cancer-associated fibroblasts. **a** UMAP visualization of 6 CAF subclusters in all (left panel), pre-RCT (middle panel), and post-RCT (right panel) samples. **b** Bar plots showing the relative proportions of 6 CAF subclusters pre- and post-RCT. **c** Box plots showing the fold change in the abundance of each CAF subcluster compared between pre- and post-RCT samples. The colors of dots and squares represent patients and sample groups, respectively. The *p* values correspond to paired Wilcoxon tests. **d** Heatmap showing the scaled expression level of signature genes in each CAF subcluster. **e** Violin plots showing the changes in the average expression of signature genesets in all CAFs between pre- and post-RCT groups (two-sided Wilcoxon test). **f** Heatmap showing the relative change in the expression level of signature genes in all CAFs and each CAF subcluster between pre- and post-RCT groups. The intensity of the color indicates the extent of upregulation in post-RCT (red) or pre-RCT (blue) samples. **g** GSVA analysis of hallmark pathways of CAFs in pre-RCT versus post-RCT samples

Fibroblasts in the tumor niche influence various processes during tumor development and influence how cancer cells respond to treatment.^28^ Therefore, we next examined the population of fibroblasts, defining six subclusters of cells: matrix cancer-associated fibroblasts (mCAFs), which highly express genes involved in the organization of extracellular matrix and epithelial proliferation; inflammatory CAFs (iCAFs), which express low levels of collagen genes but high levels of chemotactic cytokines *CXCL9*, *CXCL10*, and *CXCL12*; antigen-presenting CAFs (apCAFs), which express HLA-related genes and genes involved in leukocyte aggregation and inflammation; and vascular CAFs (vCAFs), which express angiogenesis genes *ANGPT2, COL4a1*, and *GJA4* (Fig. 5a and Supplementary Fig. 8a, b). GO term analyses showed that mCAFs were enriched for extracellular matrix organization and transforming growth factor-beta response, iCAFs were enriched for cell chemotaxis, apCAF1 was enriched for oxidative stress response, apCAF2 was enriched for epidermal development, vCAF1 was enriched for arterial development, and vCAF2 was enriched for extracellular matrix organization and cell-substrate adhesion (Supplementary Fig. 8c).

As CAFs mediate the response of cancer cells to treatment,^29^ we also compared the features of CAF subpopulations before and after RCT. We found that the relative proportion of iCAFs and a subtype of apCAFs, herein termed apCAF2, was significantly decreased, while the proportion of vCAF1 was increased by RCT (Fig. 5b, c). Evaluating the expression of signature genesets in CAF subclusters (Fig. 5d and Supplementary Fig. 8d), we found reduced angiogenic features and enhanced inflammatory scores in CAFs post-RCT (Fig. 5e, f). Specifically, the expression was lower for genesets associated with angiogenesis but higher for genesets associated with inflammation in nearly all vCAF and apCAF subclusters post-RCT (Supplementary Fig. 9). This pattern was consistent with the analysis of tumor hallmarks, which showed upregulated expression of TNF and JAK/STAT pathways and downregulated expression of angiogenesis pathway during RCT treatment (Fig. 5g). Both angiogenic and immunosuppressive processes promote tumor growth, and these data demonstrate that RCT may impair the tumor-promoting capacity of CAF populations.

### Endothelial cells show enhanced angiogenic, hypoxic, and inflammatory gene expression during RCT

**Fig. 6:**
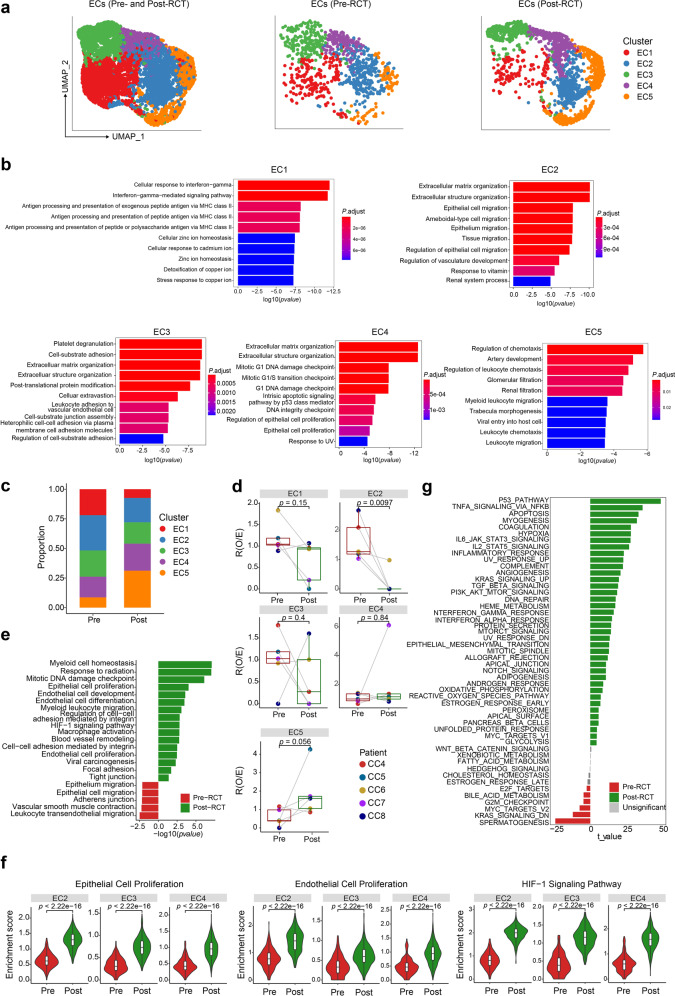
RCT enhances angiogenic, hypoxic, and inflammatory features of endothelial cells. **a** UMAP visualization of 5 EC subclusters in all (left panel), pre-RCT (middle panel), and post-RCT samples (right panel). **b** GO term analyses of EC subclusters. **c** Bar plots showing the relative proportions of 5 EC subclusters in all EC in pre- and post-RCT samples. **d** Box plots showing the fold change in the abundance of each EC subcluster compared between pre- and post-RCT samples. The colors of dots and squares represent patients and sample groups, respectively. The *p* values correspond to paired Wilcoxon tests. **e** The enriched GO terms and KEGG pathways of ECs in pre-RCT versus post-RCT samples. **f** Violin plots showing the indicated pathway enrichment of EC2-4 cells between pre- and post-RCT groups (two-sided Wilcoxon test). **g** GSVA analysis of hallmark pathways of ECs in pre-RCT versus post-RCT samples

The vascular endothelium reacts to factors that profoundly affect the growth of tumors and their response to treatment, and produces them. Here, we identified five subclusters of endothelial cells (ECs) with distinct transcriptional features (Fig. 6a and Supplementary Fig. 10a, b). GO term analyses showed that EC1 was enriched for the interferon-gamma pathway and antigen processing and presentation, EC2 was enriched for epithelial cell migration, EC3 was enriched for cell-substrate adhesion, EC4 was enriched for the DNA damage checkpoint, and EC5 was enriched for leukocyte chemotaxis (Fig. 6b).

We noticed that the proportion of EC2 cells was significantly decreased during RCT; however, EC2-4 still accounted for more than half of all ECs post-RCT (Fig. 6c, d). Indeed, consistent with the presence of pro-angiogenic CAF subclusters (Fig. 5d), the expression of genes and GO terms associated with EC development, blood vessel remodeling, and hypoxia was enriched in ECs by RCT, especially for EC2-4 (Fig. 6e, f and Supplementary Fig. 10c). Notably, in addition to enhanced angiogenesis, the expression of genes involved in epithelial proliferation was also significantly upregulated in ECs (Fig. 6e, f). Moreover, we identified enrichment of genes involved in hypoxia and angiogenesis, as well as inflammation, including TNF/NFkappaB and interleukin-related pathways, after RCT (Fig. 6g). These genes were associated with anti-tumor characteristics of lymphocytes and myeloid cells, as well as the activation of ECs by TNF and interleukins, which had been reported previously.^30^ Both angiogenic and pro-immunity features of ECs emerging in parallel complicate the interpretation of the effect of RCT on the EC populations, indicating that further studies should focus on inhibiting the pro-angiogenic side of ECs while aiming to preserve their inflammatory features during therapy.

### Clinical significance of cell subclusters and cell–cell interactions

**Fig. 7:**
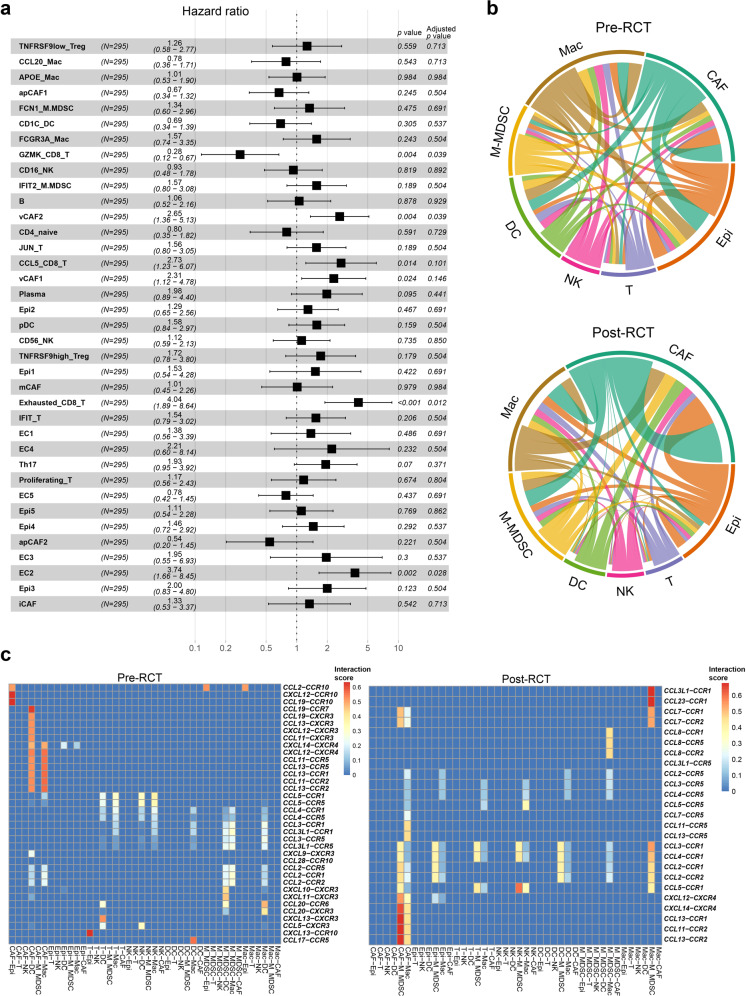
Clinical role of cell subclusters and cell-cell interactions. **a** Correlation between the abundance of cell subclusters (CIBERSORTx analysis) and patient survival in 295 TCGA bulk RNA-seq data of CC samples (Cox regression analysis). Adjusted *p* values were obtained using Benjamini & Hochberg adjustment method to control the false discovery rate for multiple comparisons. **b** Circos plot showing the cell-cell interactions in cell clusters pre- (top panel) and post-RCT (bottom panel). The thickness of each string indicates the number of different interaction pairs colored by cell clusters. **c** Summary of chemokine ligand-receptor interactions between cell clusters in pre-RCT (left panel) and post-RCT samples (right panel)

To assess the clinical role of the cell subclusters identified in our study, we used CIBERSORTx to estimate the abundance of these subclusters in the bulk RNA-seq data of 295 CC samples from TCGA, based on our scRNA-seq data. We found that vCAF2, Exhausted_CD8_T, and EC2 were significantly correlated with poor prognosis in patients with CC, while GZMK_CD8_T was significantly correlated with good prognosis (Fig. 7a).

In addition, we investigated the cell-cell interaction network in epithelial cells, NK cells, T cells, M-MDSCs, macrophages, DCs, and CAFs in pre- and post-RCT samples based on the expression of various ligand-receptor pairs (Fig. 7b and Supplementary Fig. 11). M-MDSCs, NK cells, and CAFs had more incoming interactions after RCT, while epithelial cells, T cells, and DCs had fewer incoming interactions (Fig. 7b). For outgoing interactions, M-MDSCs, NK cells, and macrophages showed fewer interactions post-RCT, while epithelial cells and CAFs showed more interactions. To investigate how immune cells were recruited, we visualized ligand-receptor interactions of chemokines between cell clusters before and after RCT (Fig. 7c). Compared to pre-RCT, there were more interactions between epithelial cells and M-MDSCs (mainly by *CCL2-CCR1/CCR2*) after RCT, as well as between CAFs and M-MDSCs (mainly by *CCL11-CCR2* and *CCL13-CCR1/CCR2*). For NK cells, a stronger interaction by *CCL5-CCR1* was seen post-RCT. Taken together, RCT affected interactions between cell clusters, which may facilitate the infiltration of immune cells into the TME.

## Discussion

Previous bulk RNA-sequencing studies of patients with CC undergoing RCT demonstrated altered gene signatures or differences in immune cell populations; however, these studies failed to provide a high-resolution assessment of gene expression patterns that would account for the heterogeneity of various cell subclusters.^9,31,32^ One study used multispectral flow cytometry to quantify lymphocyte populations in cervical smears, revealing that the number of CD8^+^ and CD4^+^ T cells declined during the first-week post-RCT but gradually increased at weeks 3 and 5.^32^ However, that study was unable to examine the T cell populations in greater depth nor investigate their functional transcriptional profiles. In addition, one clinical trial confirmed that both tumor tissue and peripheral blood mononuclear cells had fewer T cells 3 weeks after RCT.^9^ Moreover, it was shown that T cell receptors of peripheral blood mononuclear cells exhibited less diversity 3 weeks post-RCT compared to pre-RCT; however, as noted previously, the heterogeneity of various cell subclusters and their altered functional transcriptional profiles were not accounted for in that clinical trial. In the current study, we applied scRNA-seq to paired tumor samples from patients with CC to characterize the variants of epithelial, immune, and stromal cell subclusters during RCT at the single-cell level. We hoped that our findings would provide novel insights into the understanding of tumor ecosystem remodeling in response to RCT, granting clues for the design of more effective combinatory therapies capable of achieving better disease control in the long term.

One of our most striking findings was the high expression level of MCH-II genes in epithelial cells post-RCT. Studies of human small-cell lung cancer, neuroblastoma, and Merkel cell carcinoma claim that MHC-I alone is vital for the presentation of endogenous antigen by cancer cells to CD8^+^ cytotoxic T cells, suggesting that tumor elimination mainly depends on CD8^+^ cytotoxic T cells.^33^ We identified, for the first time, the upregulated expression of genes encoding MHC-II in epithelial cells of patients with CC undergoing RCT, which was expected to markedly enhance their immunogenicity. In other studies, increased MHC-II expression in tumor cells predicted the efficacy of immune checkpoint inhibitors and correlated with favorable disease prognosis.^34^ It was also reported that tumor-specific MHC-II expressed in both melanoma and breast cancer cells derived from triple-negative breast cancer patients increased tumor infiltration by lymphocytes.^35,36^ Still, there are virtually no studies indicating that RCT may increase MHC-II expression. The potentially upregulated MHC-II in some epithelial cells reported here suggests that patients with CC undergoing RCT might benefit from the well-documented ability of CD4^+^ T cells to contribute to the immune-mediated elimination of tumors.^34^ However, we did not detect accumulating CD4^+^ or CD8^+^ T cells at 3 weeks post-RCT. Therefore, future studies should incorporate multiple time points into the treatment phase to assess the role of T cell populations in the success of RCT for CC.

Along with the encouraging findings of increased MHC-II gene expression in some epithelial cells, we also uncovered evidence of a residual malignant population. Here, Epi2 expressing immunosuppressive genes (*LCN2*, *DEFB1*) survived RCT and exhibited reduced antigen presentation profiles with low lymphocyte activation potential, suggesting an immune escape that may underlie treatment resistance. Thus, scRNA-seq has revealed that the response of the epithelial cell compartment to RCT is much more complex and heterogeneous compared to how it had been understood previously. More research is needed to uncover the mechanisms underlying the balance between pro- and anti-tumoral outcomes following RCT, and to understand how this balance can be manipulated to lead to improved treatment outcomes.

MDSCs mediated the suppression of immunity by inhibiting the response of T cells, remodeling the TME, and promoting tumor angiogenesis, thus facilitating tumorigenesis.^37^ We strikingly found that FCN1^+^ M-MDSCs were significantly more abundant post-RCT and expressed higher pro-inflammatory and lower anti-inflammatory signature scores. These findings indicate a pro-inflammatory emergence of M-MDSCs post-RCT in the ecosystem of CC. Previously, Zhang et al. demonstrated that irradiation might recruit polymorphonuclear MDSCs that inhibit CD8^+^ T cell response in a mouse model of Lewis lung cancer.^38^ Those results contrast our findings, which possibly indicate the heterogeneity in the TME between different tumor types during RCT treatment as well as the presence of potentially distinct therapeutic targets in various settings. In addition, our study revealed that the expression of genes involved in antigen processing and presentation in DCs was also significantly enhanced during RCT treatment. Overall, the myeloid cell compartment demonstrated a coherent transcriptional program promoting leukocyte migration and adhesion, as well as innate immune activation, during treatment. Thus, RCT has a significant regulatory role in shaping the tumor immune response due to its effects on the subsets of myeloid cells.

We also observed changes in the relative proportions and transcriptional characteristics of T and NK cell subclusters following RCT. Interestingly, one of the most striking changes was attributed to NK cells rather than T cells, which had not been previously reported. We found that CD16^+^ NK cells with an enriched cytotoxicity signature and higher expression of genes involved in leukocyte migration and adhesion were significantly increased during RCT. On the contrary, the number of CD56^+^ NK cells decreased during treatment, which indicates their low functional status. Although a recent study has demonstrated that CD56 can stimulate the cytotoxic functions of NK cells through the non-receptor tyrosine kinase Pyk2,^39^ CD56dim CD16^+^ NK cells appear to be more cytotoxic than CD56bright NK cells, as CD16 can mediate antibody-dependent cell-mediated cytotoxicity.^40^ This is consistent with our results of CD16^+^ NK cells having an enriched cytotoxic signature (*PRF1, NKG7*, and *GZMB*). Moreover, high expression of genes, including *PRF1, NKG7, GZMB, GZMH*, and *IFNG*, was associated with better survival, which similarly indicates the probable functional significance of CD16^+^ NK cells in this setting. Using cells from a patient-derived xenograft model in dogs and a canine sarcoma model, Canter et al. showed that radiotherapy could enhance NK cell (CD56dim, NKp46^+^) cytotoxicity ex vivo.^41^ In conclusion, RCT seems to have an effect on the TME of CC, potentially regulating the composition of immune cells, including markedly increased frequency of CD16^+^ NK cells, which could augment the anti-tumor response.

In our study, we revealed that RCT treatment caused a significant increase in the proportions of naïve CD4^+^ cells and a decrease in the proportions of exhausted CD8^+^ T cells. Naïve T cells have a positive impact on anti-cancer immunity, replenishing the T cell population of the TME. However, a recent study has demonstrated that blocking the recruitment of naïve CD4^+^ T cells can reverse the immunosuppression by Treg.^42,43^ Further, that study showed that the abundance of naïve CD4^+^ T cells and Tregs was closely correlated and that both indicated a poor prognosis for patients with breast cancer.^42^ Thus, targeting naïve CD4^+^ T cells may enhance the efficacy of RCT in CC, which is a possibility warranting further investigation. At the post-RCT time point, we also observed a weakening of inhibitory features in exhausted CD8^+^ T cells, manifested as a decrease in the expression of *HAVCR2* and *TIGIT*. However, the cytotoxic features of T cell subclusters did not increase during RCT, unlike what was observed in NK cell clusters. Thus, 3 weeks after the start of RCT, there is evidence of predominantly innate immune activation, as in M-MDSCs and NK cells, as opposed to adaptive immune activation evidenced by an attenuated immune response of T cells. It is not yet clear whether RCT would stimulate adaptive immune responses before or after 3 weeks, and further research is needed to conduct an appropriate investigation.

While the abundance of some EC subclusters was reduced by RCT, we also found enriched expression of genes involved in epithelial cell proliferation, EC proliferation, and the HIF-1 signaling pathway. Taken together, the transcriptional data indicate that ECs modify the extracellular matrix and promote angiogenesis, while upregulating the immune activation during treatment. As angiogenesis plays a pivotal role in tumor recurrence after treatment,^44^ anti-angiogenic therapies can be a valuable addition to the treatment regimen of patients with CC undergoing RCT.

In conclusion, we present a comprehensive transcriptional landscape of tumor ecosystem remodeling induced by RCT. Our study provides a detailed picture of the delicate balance between pro- and anti-tumor programs and cell subclusters that remain present in tissues 3 weeks after RCT. This study identifies potentially important novel players in the post-RCT ecosystem. We show that RCT leaves behind a population of epithelial cells with upregulated expression of genes encoding MHC class II molecules, as well as a separate population of malignant epithelial cells. We also demonstrate that RCT elicits anti-tumor immune responses, including the recruitment of CD16^+^ NK cells with higher cytotoxic gene expression and FCN1^+^ M-MDSC cells with increased pro-inflammatory features. Future studies with larger samples are needed to validate our results and investigate cell subclusters associated with the treatment response. Taken together, we provide unprecedented insight into the tumor ecosystem remodeling induced by RCT, which will help optimize and improve treatment strategies for CC.

## Materials and methods

### Patients and sample collection

This study was approved by the Ethics Committee of Shandong Cancer Hospital and Institute (SDTHEC201906009). A total of seven patients (median age, 50.5 years; range, 38–62 years) were enrolled in the study, and written informed consent was obtained. All patients had an HPV infection. Five patients were diagnosed with cervical squamous cell carcinoma at a locally advanced stage and received RCT. Tumor samples were collected by biopsy 1 week before and 3 weeks after the initiation of RCT. Radiotherapy consisted of pelvic external beam radiation therapy (27–30 Gy; 15–17 fractions) and brachytherapy (5–10 Gy; 1–2 fractions), accompanied by one cycle of chemotherapy with paclitaxel (175 mg/m^2^) followed by nedaplatin (75–80 mg/m^2^). All patients showed a partial response (67–90% decrease in tumor size) to RCT. Two additional patients were diagnosed with cervical intraepithelial neoplasia; they underwent cervical loop electrosurgical excision. Normal tissue samples were collected during surgery. To confirm our scRNA-seq results, we also analyzed 15 pairs of pre- and post-RCT formalin-fixed paraffin-embedded (FFPE) samples of CC (validation cohort 1) and 20 pairs of pre- and post-RCT bulk RNA-seq data samples of CC that had been published previously (validation cohort 2).^45^

### Sample processing and cell sorting

Tumor biopsy samples were immediately transported to the laboratory for digestion. After washing with phosphate-buffered saline (PBS; Gibco), each sample was cut into pieces of ~1 mm^3^ and digested with 5 ml of collagenase IV (2 mg/mL, Sigma) and DNase I (1 mg/mL, Sigma) solution in a 37 °C incubator for 30 min. Next, the sample was mixed with 3 ml 1% BSA and filtered through a 70-µm mesh size cell strainer (Corning). After centrifugation (300 × *g*, 5 min, 4°C), the supernatant was discarded, and the cell pellet was resuspended and incubated (5 min, 4 °C) in 1.5 ml 1× erythrocyte lysis buffer (Biolegend). The sample was then centrifuged again (300 × *g*, 5 min, 4 °C), and the cell pellet was resuspended in 1 ml 1% BSA and incubated with antibodies recognizing EpCAM (BD Biosciences), CD45 (BD Biosciences), and CD31 (BD Biosciences) in the dark (15 min, 4 °C). After washing twice with 1 ml 1% BSA, the cells were resuspended in 100 µl 1% BSA and incubated with 7-AAD Viability Staining Solution (eBioscience) for 3 min before sorting using the BD FACSAria II flow cytometer (BD Biosciences). Isolated cells were then subjected to single-cell library preparation and sequencing.

### scRNA-sequencing, pre-processing, and integration of datasets

Single-cell transcriptome amplification and library preparation were performed using the Single-Cell 3’ Library Kit v3 (10× Genomics) according to the manufacturer’s guidelines. Libraries were pooled and sequenced across six lanes in the Illumina NovaSeq 6000 system (Illumina, Inc., San Diego, CA, USA). Transcriptomic data were generated by 10× Genomics and subsequently aligned and quantified using CellRanger (version 4.0.0) with default parameters. Each batch of data was subjected to Seurat (version 3.2.0), and doublets were identified using DoubletFinder (version 1.0.1) with default parameters. After removing the doublets, data from cells expressing <200 genes and containing >20% of mitochondrial gene reads were categorized as low-quality and subsequently removed. The remaining data were used for integration with the “IntegrateData” function in Seurat and dims of 1:30. The second quality control step was subsequently performed, setting the number of UMI to <100,000 and the number of genes to <7500. Data from 60,550 cells with a mean expression of 2730 genes were subjected to further analyses.

### Cell type identification and dimensionality reduction

Based on the results of the “integrated” assay, the data were scaled according to the genes expressed in cells, with the exception of those whose expression positively correlated with the mean expression of cell cycle genes, including *UBE2C*, *HMGB2*, *HMGN2*, *TUBA1B*, *MKI67*, *CCNB1*, *TUBB*, *TOP2A*, and *TUBB4A*. For the dimensionality reduction, highly variable genes were used for principal component (PC) detection, with the selected PCs further used for the Uniform Manifold Approximation and Projection analysis. Cells were subsequently clustered with a “resolution” set to 0.9, which identified nine main clusters. DEGs were screened using the “FindAllMarkers” function, with the parameter min.pct of 0.25. These clusters were annotated according to the expression of lineage-specific genes in the “RNA” assay. Analyses for each cell type and detection of subclusters were performed using a similar strategy.

### Relative fold changes in cell clusters

As described in the previous study,^46^ the ratio (R) of observed (O) to random expected (E) cell number could adjust cell sampling bias for each patient, indicating cluster enrichment in a particular sample. The R(O/E) for each cluster in distinct samples was calculated using the Chi-square test. The value of R(O/E) > 1 indicated the enrichment of the cell cluster in the sample. Differential abundance analysis for cell subclusters was performed by paired Wilcoxon tests.

### GO and KEGG enrichment and GSVA

The top 50 DEGs identified in different clusters and subclusters were used for GO and Kyoto Encyclopedia of Genes and Genomes (KEGG) enrichment via the clusterProfiler package (version 3.14.3). Five hundred randomly selected cells per cluster were sampled, and the count matrix was extracted for the GSVA. GSVA for hallmark genes was performed using files downloaded from MSigDB (https://www.gsea-msigdb.org/gsea/msigdb/), with parameters set as mx.diff = FALSE and kcdf = “Poisson”.

### CNA prediction

The count matrix extracted from the Seurat object was subjected to Copy Number Alteration (CNA) prediction using Copycat (version 1.0.5), with key parameters set as ngene.chr = 5, win.size = 25, KS.cut = 0.15, and distance = “euclidean”.

### Cell cycle anticipation and scoring for genesets

A core set of G1/S and G2/M genes reported before^47^ was subjected to “CellCycleScoring” to predict the phase of the cell cycle. Geneset scoring was performed using the “AddModuleScore” function in Seurat. The genes used for scoring in Epi (Fig. 2 and Supplementary Fig. S3) were extracted from GO terms enriched in specific pathways. Scoring for lymphoid features was performed based on predefined marker genes for naïve T cells (*CCR7*, *TCF7*, *LEF1*, *SELL*), cytotoxic cells (*GZMK*, *IFNG*, *GZMH*, *GNLY*, *PRF1*, *NKG7*, *GZMA*, *GZMB*), inhibitory T cells (*LAG3*, *PDCD1*, *CTLA4*, *TIGIT*, *BTLA*, *HAVCR2*), Treg cells (*IL2RA*, *FOXP3*, *IKZF2*), and proliferating cells (*MKI67*, *CDK1*, *STMN1*). Genes for scoring myeloid clusters (M1 and M2) were predefined on the basis of published literature.^48^

### The abundance and clinical significance of cell subclusters in bulk RNA-seq data

Bulk RNA-seq data of 295 CC samples were downloaded from TCGA (http://www.cbioportal.org/) using the “getProfileData” function in the cgdsr package, with the “geneticProfiles” parameter set as “cesc_tcga_rna_seq_v2_mrna”. Deconvolution was performed using CIBERSORTx^49^ algorithm with top 50 DEGs of each subcluster to infer the composition of each subcluster in the 295 bulk RNA-seq data of CC samples downloaded from TCGA. The hazard ratios of subclusters were then calculated using a multi-factor Cox regression model with the composition obtained as a result of deconvolution.

### Differential expression changes

As a simple way to quantify changes in the expression of specific genes in each cluster, the average fold change in expression (post-RCT vs. pre-RCT) was calculated. The Wilcoxon rank sum test was performed, and the adjusted *p* value (*p*.adjust) was obtained after the Bonferroni correction. For genes that showed relatively high expression pre-RCT, the *p*.adjust values were multiplied by −1. Consequently, *p*.adjust was visualized in heatmaps as -log10 (*p*.adjust).

### Immunohistochemistry

Paired pre- and post-RCT FFPE tissues collected from 15 patients with CC were used for IHC. The following antibodies were applied to detect the respective proteins: Anti-CD16 (Cat# ab246222, Abcam), Anti-FCN1 (Cat#ab223712, Abcam), and Anti-HLA-DR + DP + DQ (Cat# ab7856, Abcam). Visualization was performed using Zeiss (AxioScan. Z1) at ×200 magnification, and positively stained cells were counted using a computerized image analysis system (Image J v1.53). Two experienced pathologists independently performed all IHC evaluations.

### Cell-cell interactions

The cell-cell interaction network in epithelial cells, NK cells, T cells, M-MDSCs, macrophages, DCs, and CAFs in pre- and post-RCT samples were predicted using CellCall^50^ with default parameters. Ligand-receptor pairs enriched between clusters were shown in pre-RCT and post-RCT samples.

## Supplementary information

Supplementary Materials

Supplementary Fig. 1

Supplementary Fig. 2

Supplementary Fig. 3

Supplementary Fig. 4

Supplementary Fig. 5

Supplementary Fig. 6

Supplementary Fig. 7

Supplementary Fig. 8

Supplementary Fig. 9

Supplementary Fig. 10

Supplementary Fig. 11

## Data Availability

Raw and processed scRNA-seq data of three normal cervical samples and five post-RCT advanced-stage CC samples were uploaded to the Genome Sequence Archive (GSA) of the National Genomics Data Center (PRJCA011382), which are publicly accessible at https://ngdc.cncb.ac.cn/gsa-human and https://ngdc.cncb.ac.cn/omix, respectively. Raw and processed scRNA-seq data of five pre-RCT advanced-stage CC samples had been previously deposited and publicly accessible in the GSA (PRJCA008573). The data analysis script is available from the corresponding author upon reasonable request.
