# Supplementary material for: Single-cell RNA-sequencing reveals radiochemotherapy-induced innate immune activation and MHC-II upregulation in cervical cancer
Source: Signal Transduct Target Ther. 2023 Jan 30;8:44. doi: 10.1038/s41392-022-01264-9 (PMC9884664; doi:10.1038/s41392-022-01264-9)
Supplement: Supplementary file 1 — Supplementary Materials [file 41392_2022_1264_MOESM1_ESM.docx]

Supplementary Materials for

Single-cell RNA sequencing reveals radiochemotherapy-induced innate immune activation and MHC-II upregulation in cervical cancer

Chao Liu^1,2^, Xiaohui Li^1^, Qingyu Huang^1^, Min Zhang^3^, Tianyu Lei^4^, Fuhao Wang^5^, Wenxue Zou^1^, Rui Huang^1^, Xiaoyu Hu^1^, Cong Wang^6^, Xiaoling Zhang^6^, Bing Sun^7^, Ligang Xing^1,2*^, Jinbo Yue^1,2*^, Jinming Yu^1,2*^

Correspondence to: [sdyujinming@163.com](mailto:sdyujinming@163.com); [jbyue@sdfmu.edu.cn](mailto:jbyue@sdfmu.edu.cn); [xinglg@medmail.com.cn](mailto:xinglg@medmail.com.cn)

**This PDF file includes:**

Supplementary Fig. 1 to Supplementary Fig. 11


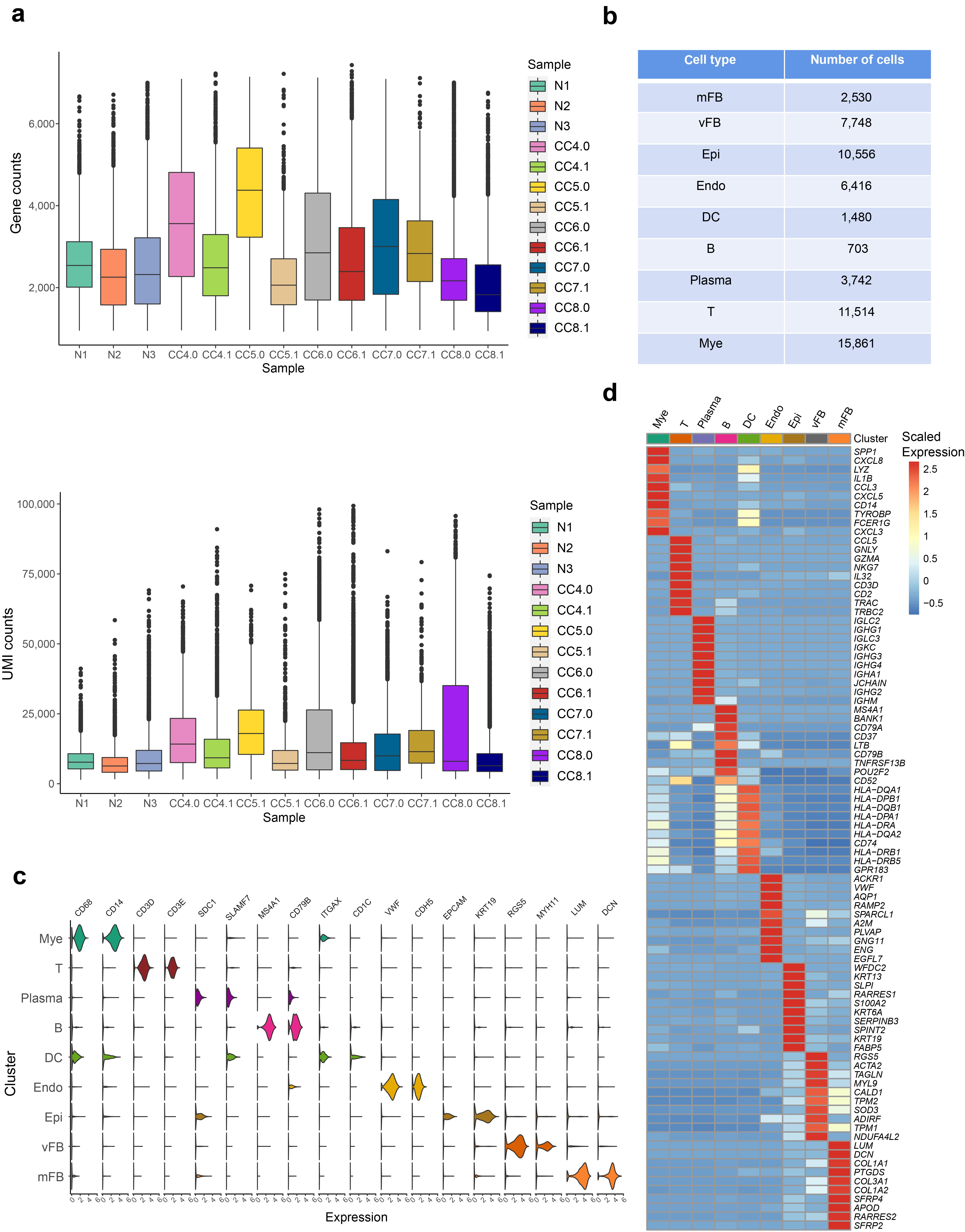


**Supplementary Fig. 1. Quality control and identification of main cell types in scRNA-seq data.**

1. Box plots displaying gene counts (top panel) and UMI counts (bottom panel) in each sample of scRNA-seq data.
2. The number of analyzed cells in each cell population.
3. Violin plots showing an overview of expression of known marker genes in cell clusters.
4. Heatmap showing the relative expression level of the top 10 DEGs in 9 main cell clusters.


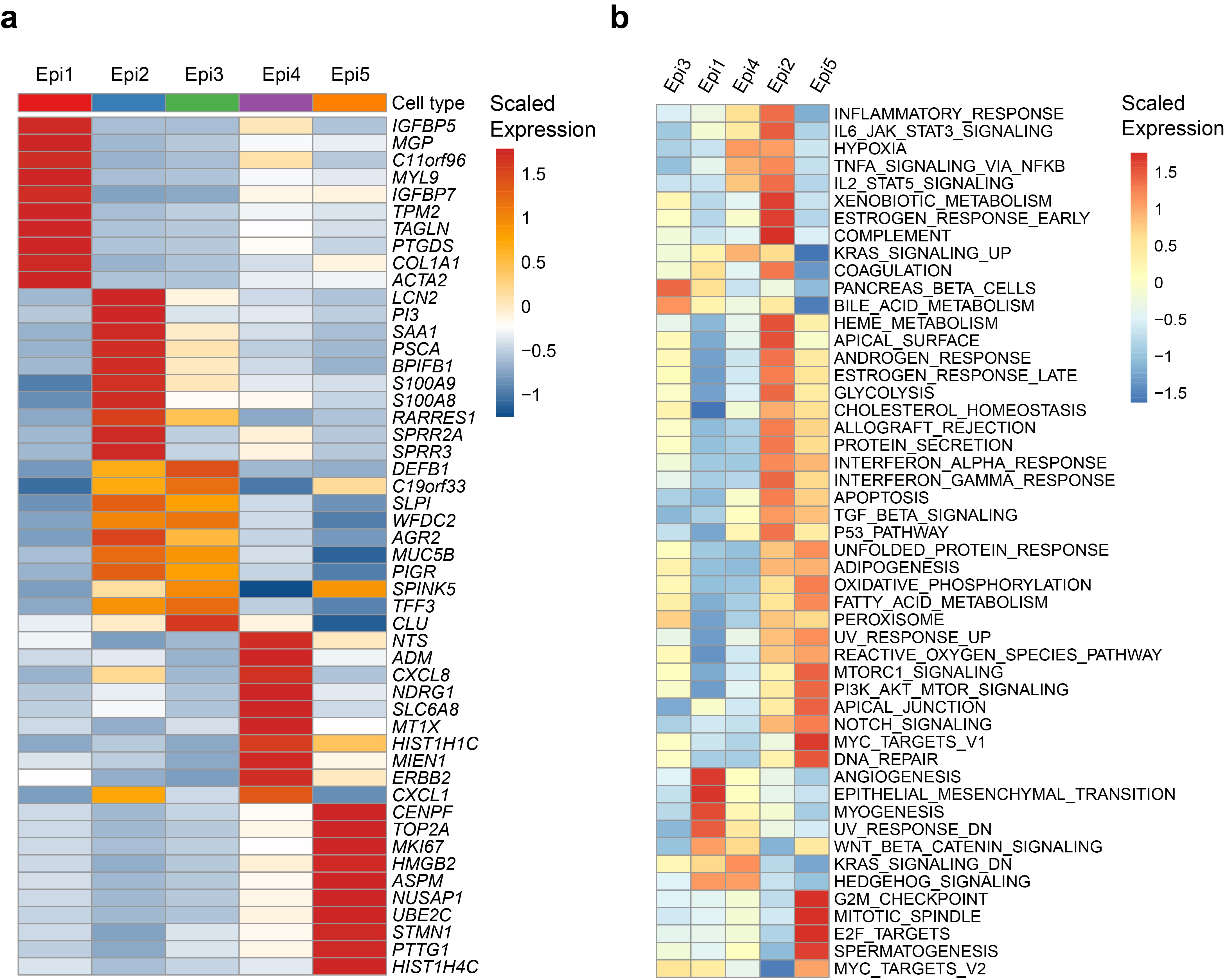


**Supplementary Fig. 2. Transcriptomic features of 5 epithelial cell subclusters.**

1. Heatmap showing the relative expression level of the top 10 DEGs in 5 Epi subclusters.
2. Heatmap showing GSVA analysis of pathway activities for 5 subclusters.


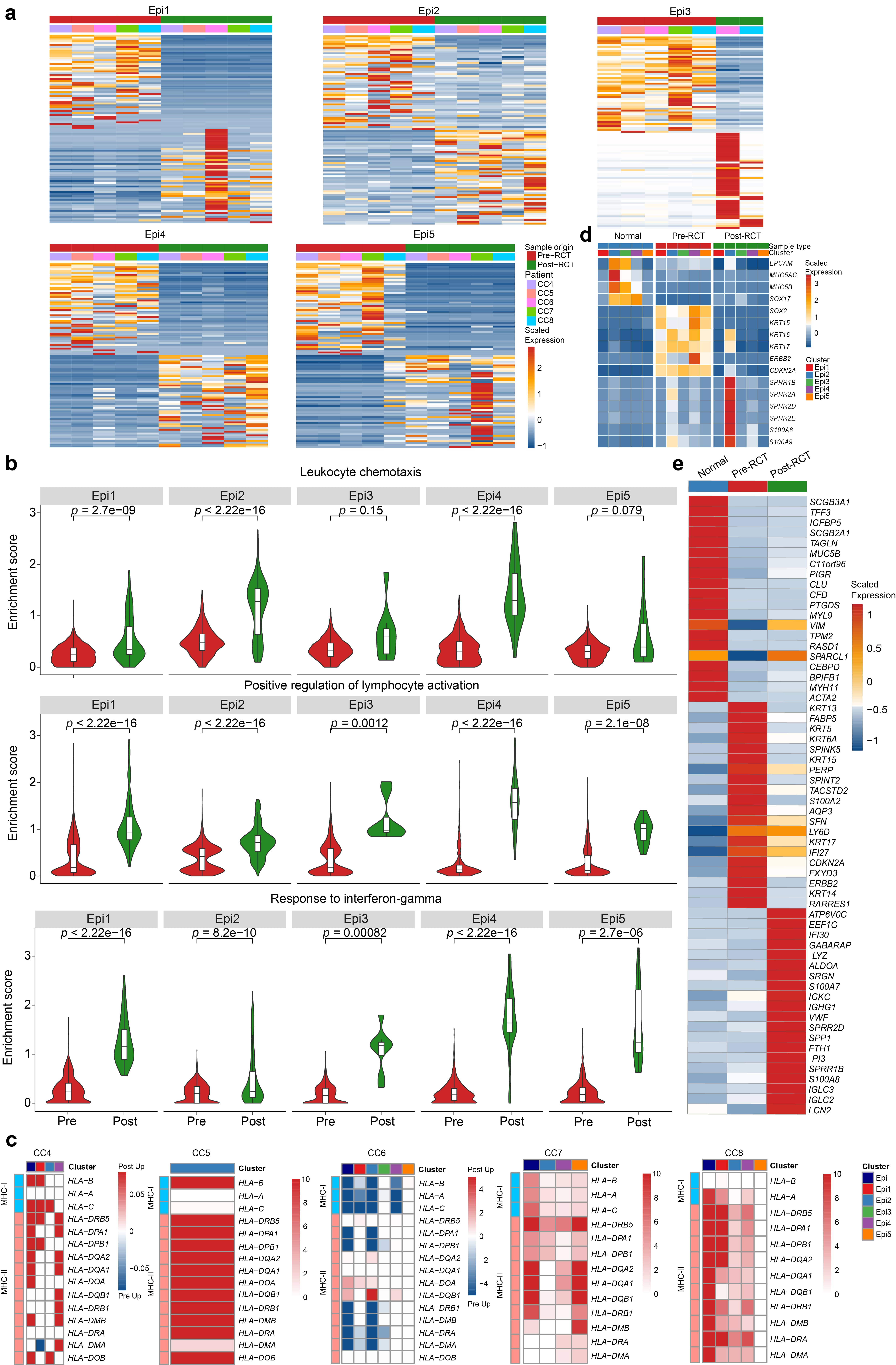


**Supplementary Fig. 3. Pre- vs. post-RCT comparison of transcriptomic features in 5 epithelial cell subclusters.**

1. Heatmaps showing the relative expression level of the top 50 DEGs of each Epi cluster between the pre- and post-RCT groups. The data for Epi3 in 3 post-RCT samples were missing because there was no Epi3 detected in the 3 samples.
2. Violin plots showing the indicated pathway enrichment of 5 EC subclusters between pre- and post-RCT samples (two-sided Wilcoxon test).
3. Heatmap showing the relative changes in MHC class I and II gene expression in pre- and post-RCT samples of each patient. The data for some Epi subclusters were missing due to the post-RCT cell number of < 2. The intensity of the color indicates the extent of upregulation in post-RCT (red) or pre-RCT (blue) samples.
4. Heatmap showing the expression level of representative genes for each Epi subcluster in normal, pre-, and post-RCT samples.
5. Heatmap showing the expression level of the top 20 DEGs in epithelial cells in normal, pre-, and post-RCT samples.


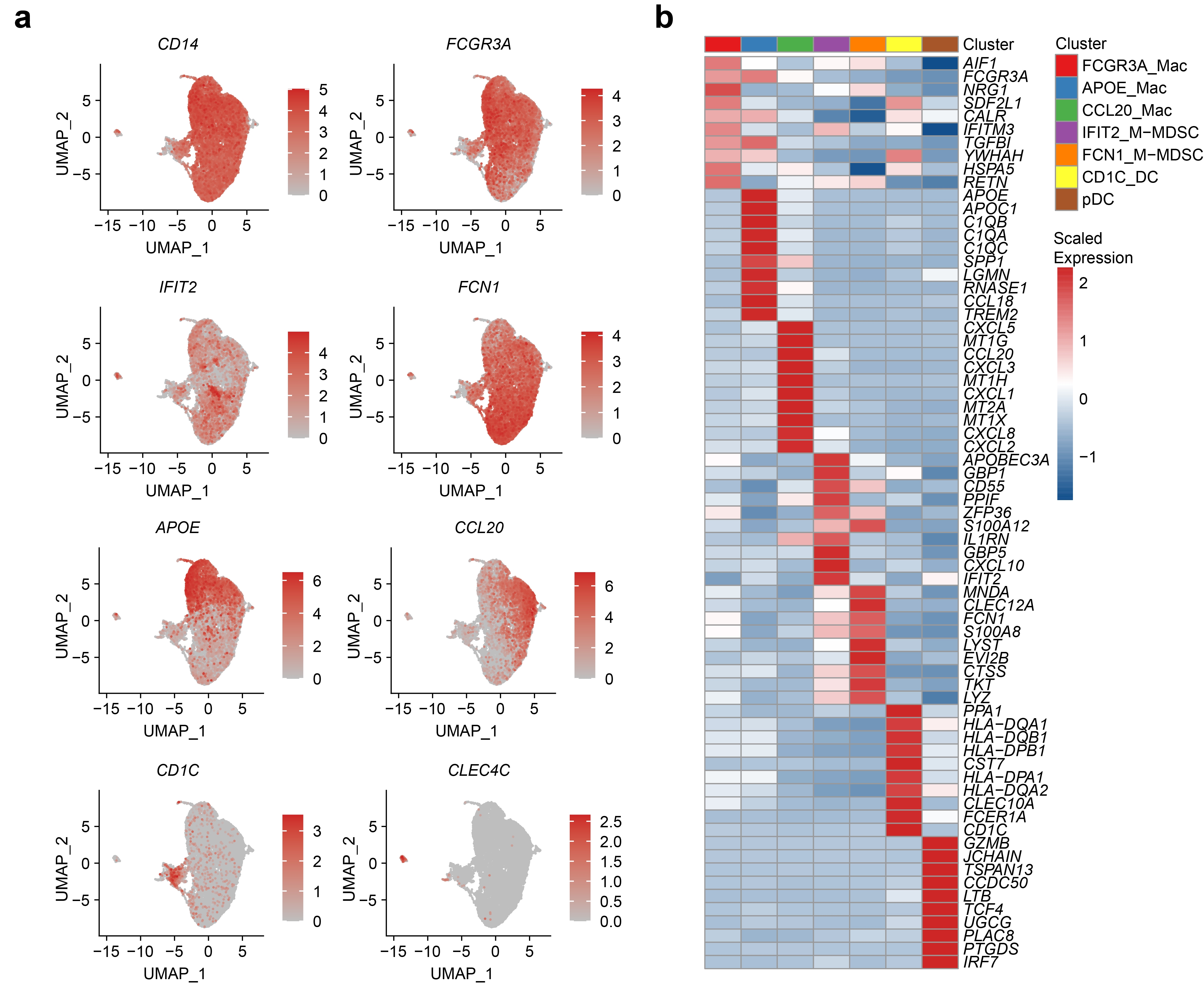


**Supplementary Fig. 4. Identification of myeloid cell subclusters.**

1. UMAP visualization of the expression of known marker genes in myeloid cell subclusters.
2. Heatmaps showing the relative expression level of the top 10 DEGs in each myeloid subcluster.


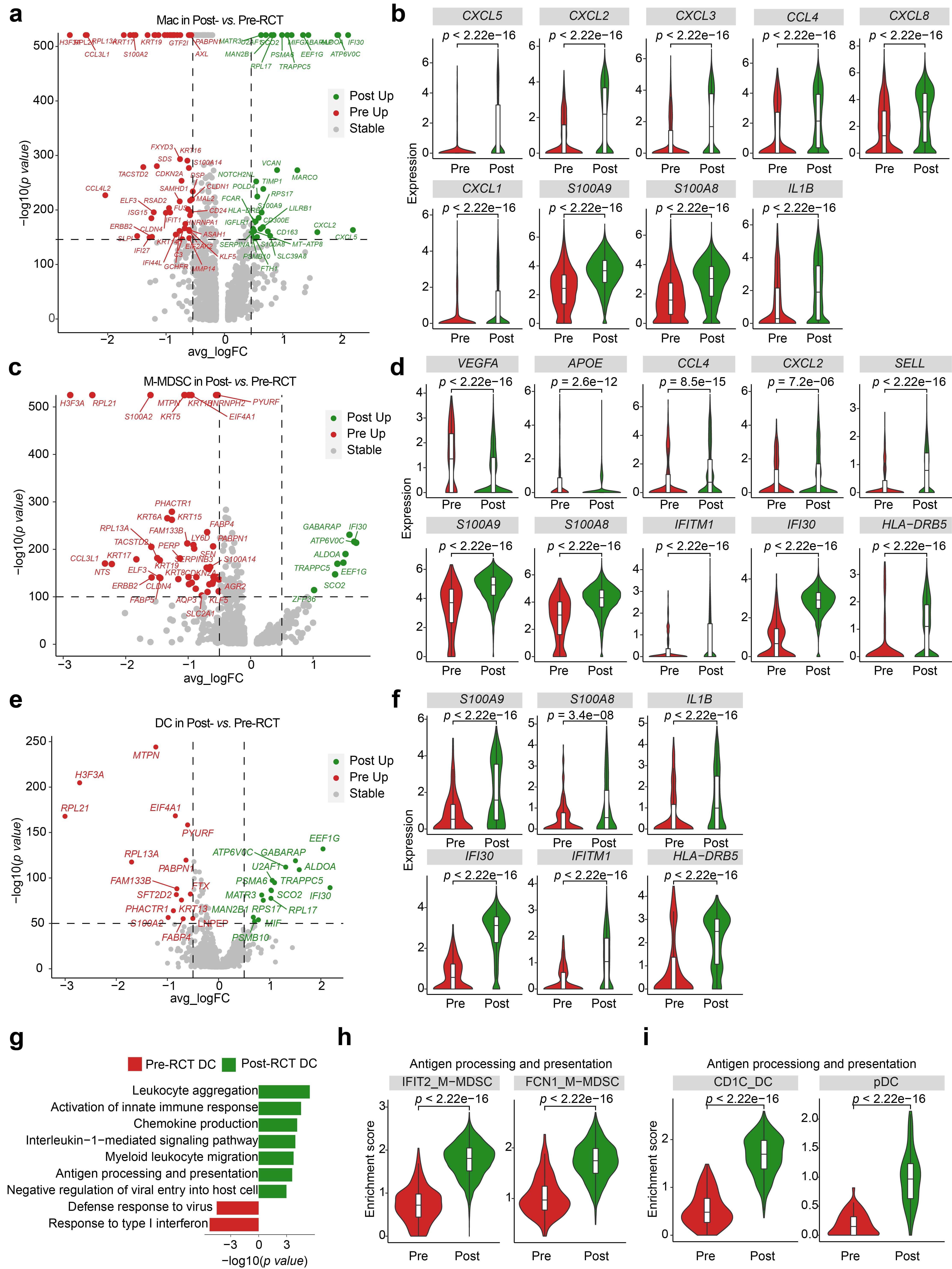


**Supplementary Fig. 5. Impact of RCT on macrophages, M-MDSCs, and DCs.**

1. Volcano plot showing the differential expression of genes in macrophages between pre- and post-RCT groups. The colored dots represent the top most variable genes.
2. Violin plots showing the expression of the indicated DEGs in macrophages from pre- and post-RCT samples.
3. Volcano plot showing the differential expression of genes in M-MDSC cells between pre- and post-RCT groups. The colored dots represent the top most variable genes.
4. Violin plots showing the expression of indicated DEGs in M-MDSC cells in pre- and post-RCT samples.
5. Volcano plot showing the differential expression of genes in DC between pre- and post-RCT groups. The colored dots represent the top most variable genes.
6. Violin plots showing the expression of indicated DEGs in DCs pre- and post-RCT.
7. GO term analysis of DCs in pre- versus post-RCT samples.
8. Violin plots showing the indicated pathway enrichment of 2 M-MDSC subclusters between pre- and post-RCT groups.
9. Violin plots showing the indicated pathway enrichment of 2 DC subclusters between pre- and post-RCT groups.

All statistical analyses in the figure are two-sided Wilcoxon test.


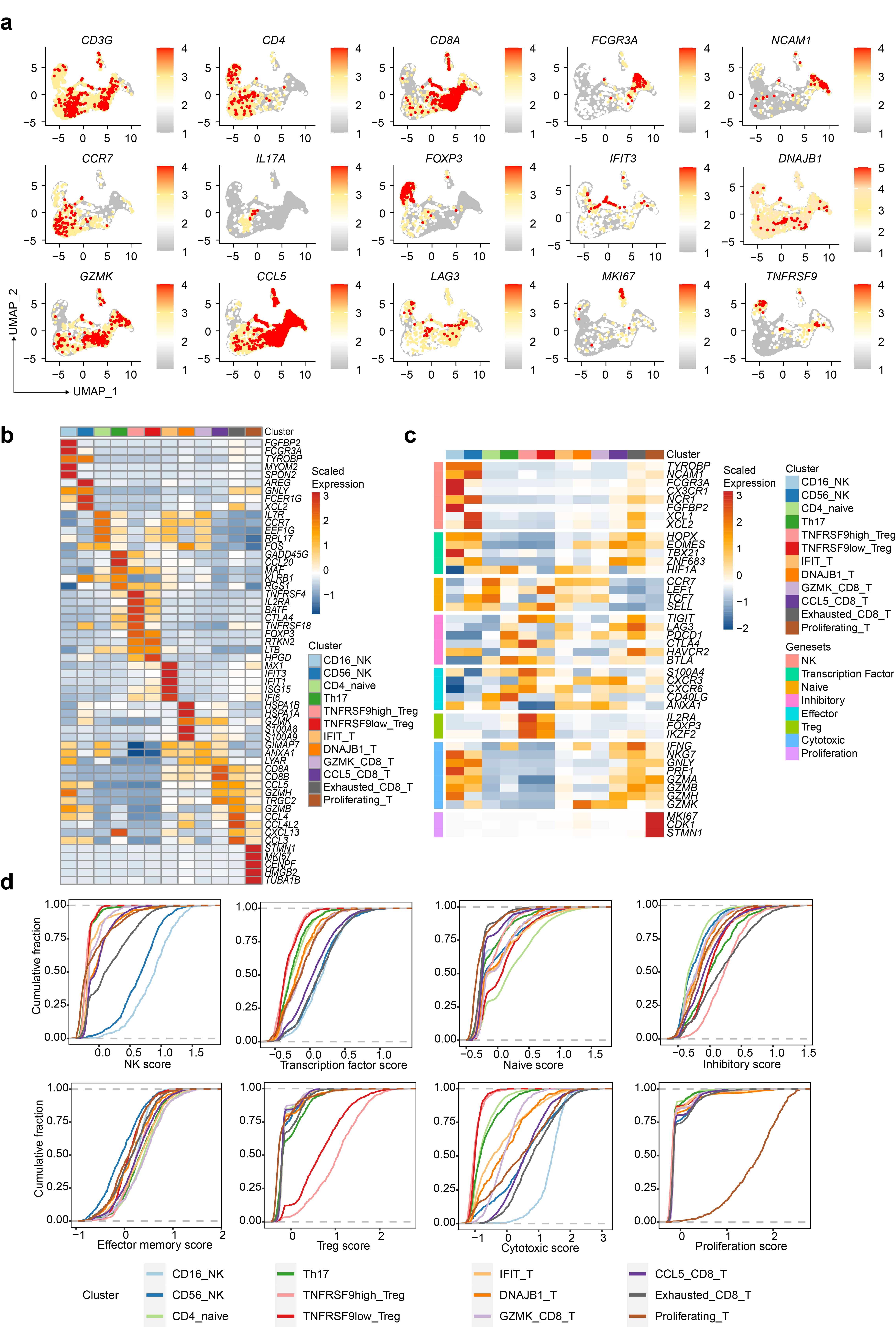


**Supplementary Fig. 6. Identification of 12 lymphocyte subclusters.**

1. UMAP visualization of the expression of known marker genes in lymphocyte subclusters.
2. Heatmap showing the relative expression level of the top 5 DEGs in each lymphocyte subcluster.
3. Heatmap showing the relative expression level of signature genes in each lymphocyte subcluster.
4. Cumulative fraction analysis showing the scoring curves of signature genesets in each lymphocyte subcluster.


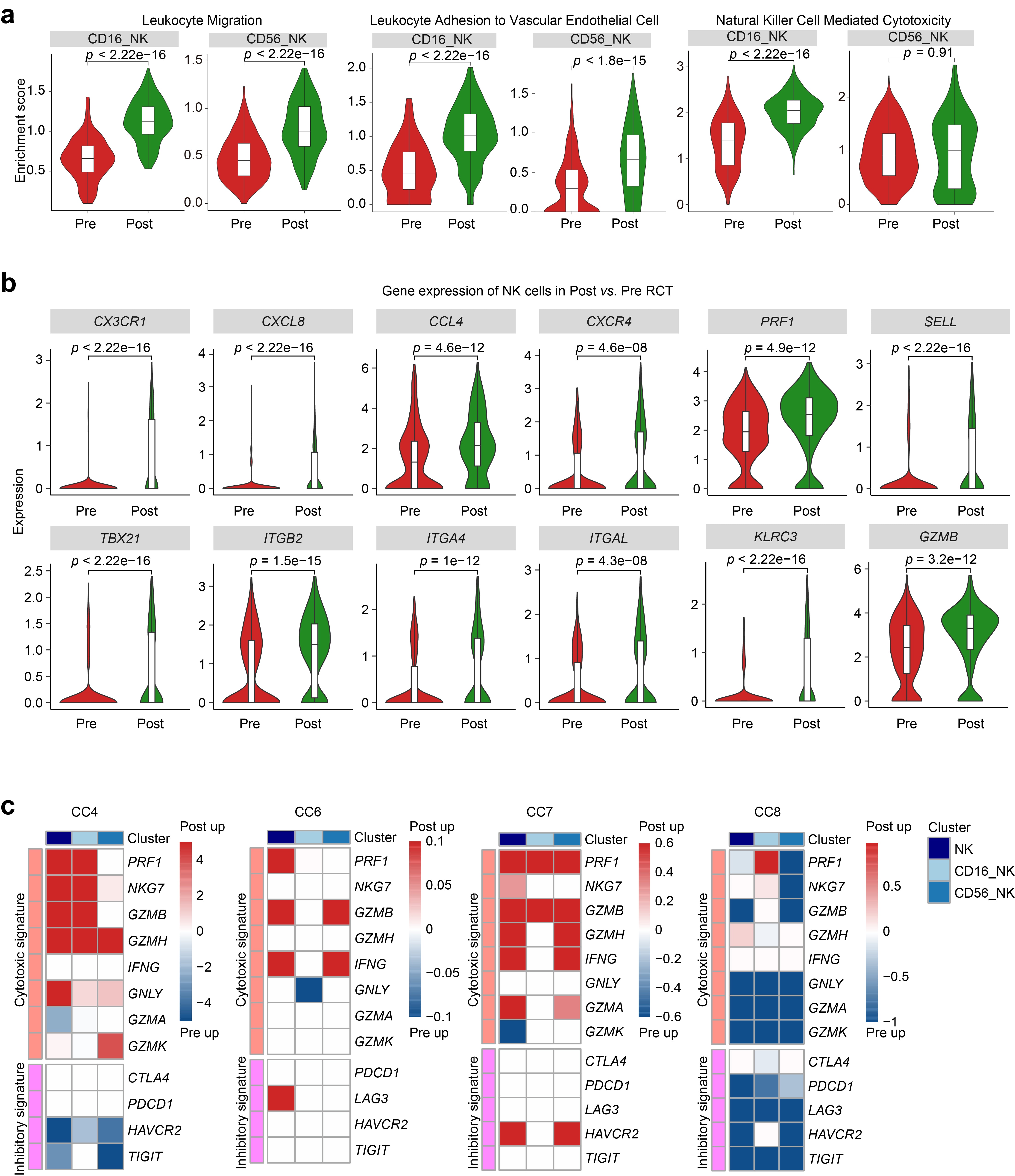


**Supplementary Fig. 7.** Transcriptional changes in NK cells pre- and post-RCT.

1. Violin plots showing the indicated pathway enrichment of 2 NK subclusters between pre- and post-RCT groups.
2. Violin plots showing the expression of indicated DEGs of NK cells between pre- and post-RCT groups.
3. Heatmap showing the relative changes in cytotoxic signature gene expression in NK cells between pre- and post-RCT samples of each patient. The data from CC5 were missing because there was no NK cells in the pre-RCT sample. The intensity of the color indicates the extent of upregulation in post-RCT (red) or pre-RCT (blue) samples.

All statistical analyses in the figure are two-sided Wilcoxon test.


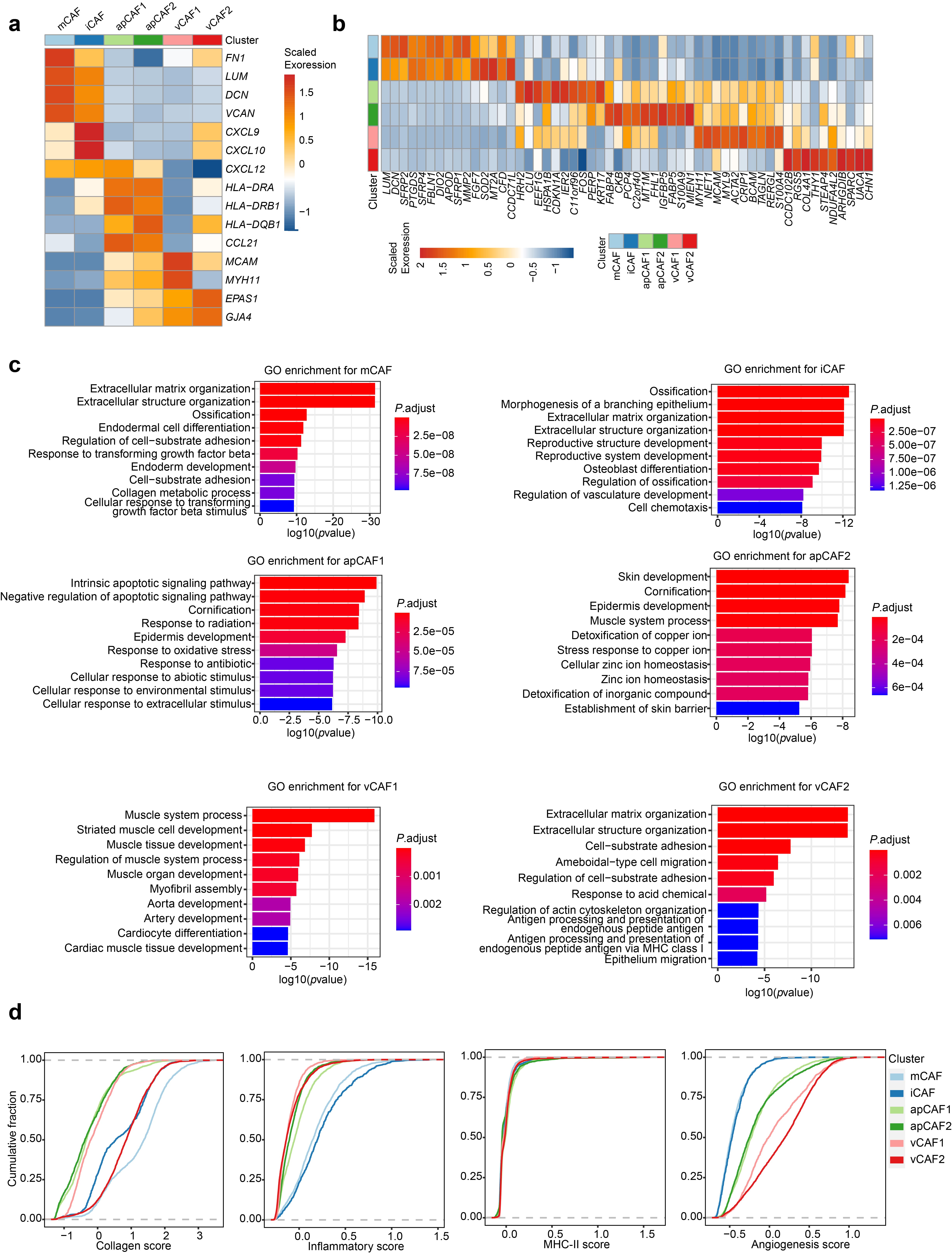


**Supplementary Fig. 8. Transcriptomic features of cancer-associated fibroblast subtypes and changes in their gene expression post-RCT.**

1. Heatmap showing the scaled expression level of known marker genes in each CAF subcluster.
2. Heatmap showing the relative expression level of the top 10 DEGs in CAF subclusters.
3. GO term analyses of CAF subclusters.
4. Cumulative fraction analysis showing the scoring curves of signature genesets in each CAF subcluster.


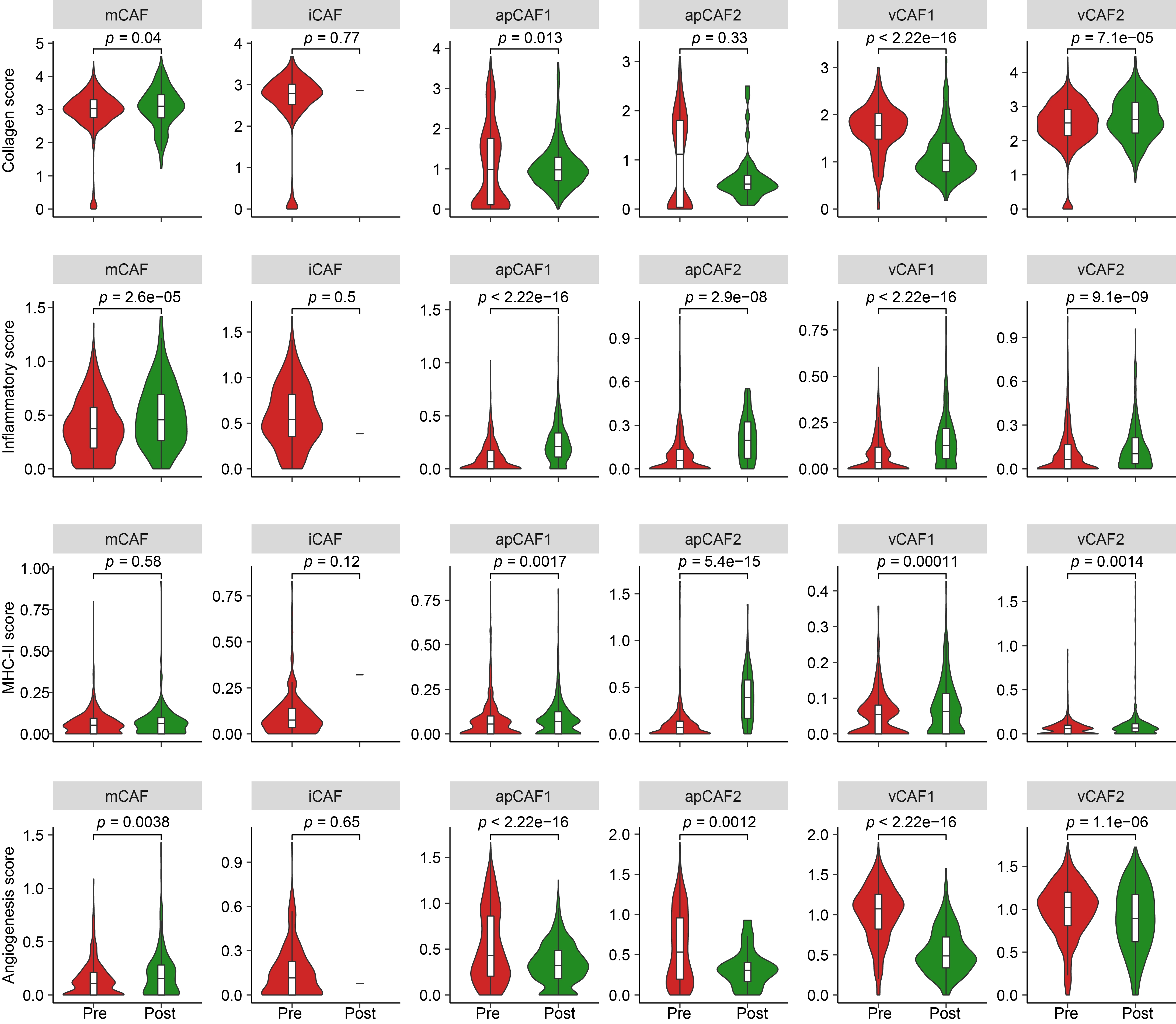


**Supplementary Fig. 9. Changes in the average expression of signature genesets in each CAF subcluster between pre- and post-RCT groups (two-sided Wilcoxon test).**


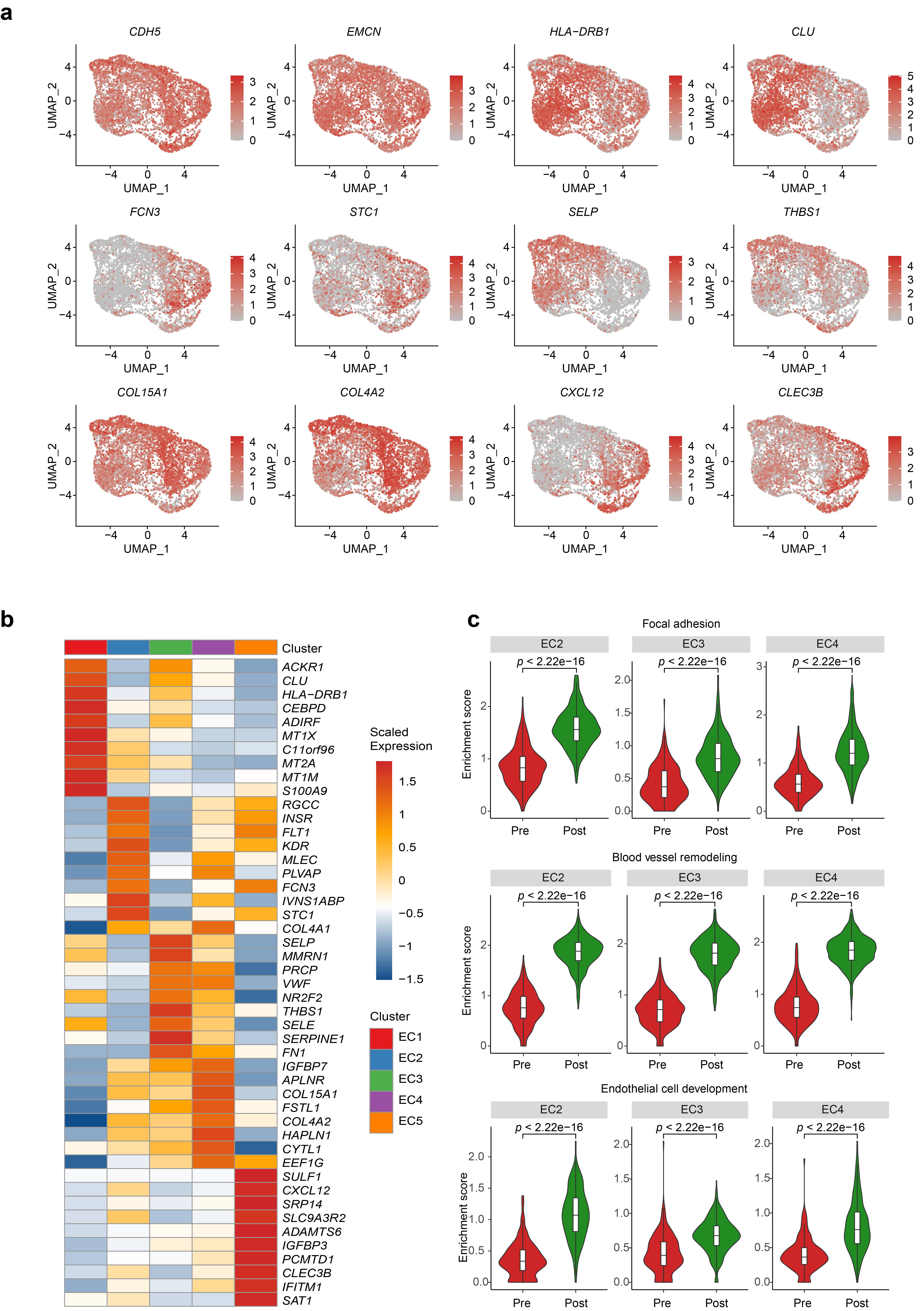


**Supplementary Fig. 10. Identification of endothelial subclusters and changes in their gene expression post-RCT.**

1. UMAP visualization of the expression of known marker genes in ECs.
2. Heatmap showing the relative expression level of the top 10 DEGs of EC subclusters.
3. Violin plots showing specific pathway enrichment of EC2/3/4 subclusters between pre- and post-RCT groups (two-sided Wilcoxon test).


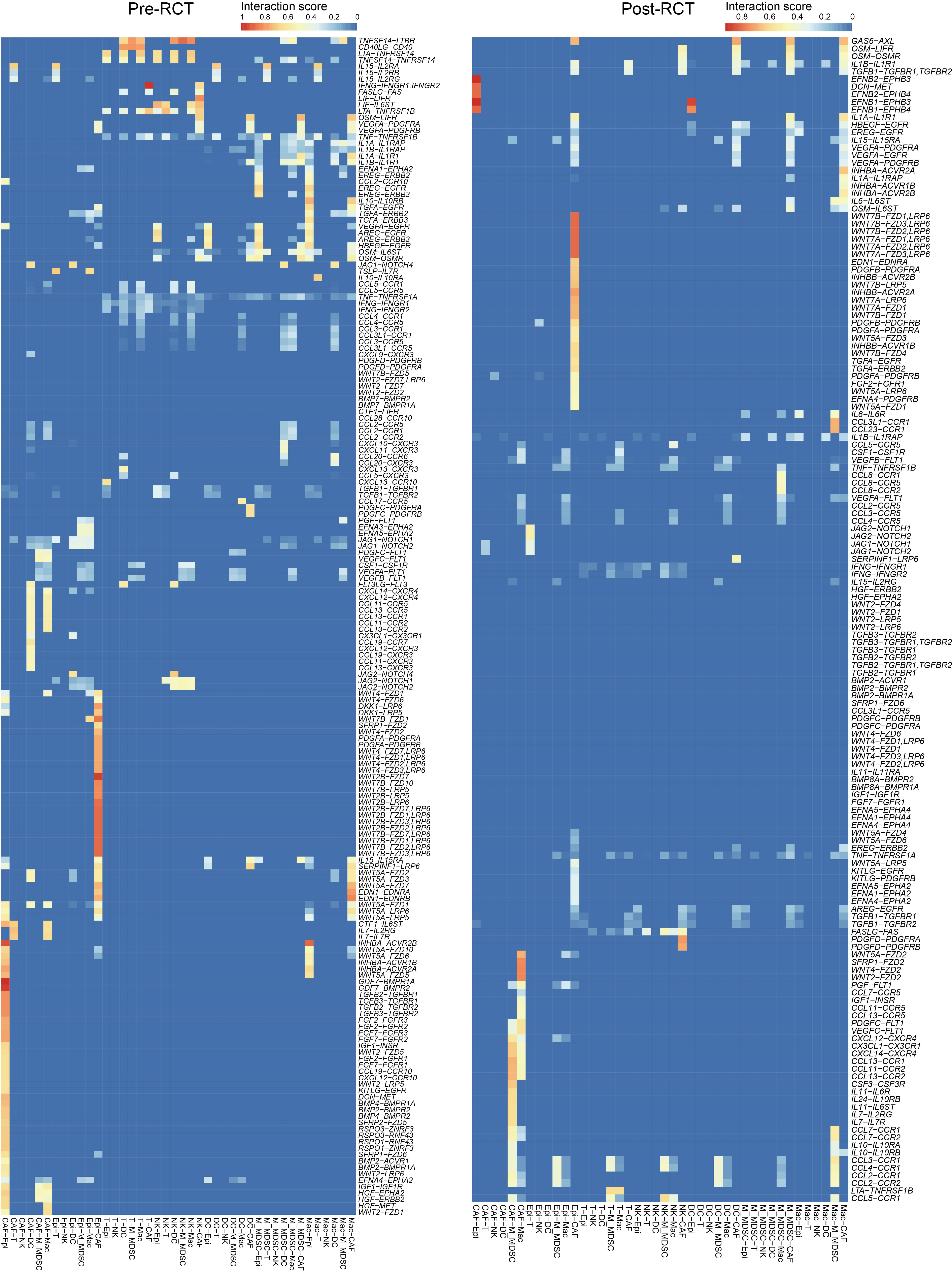


**Supplementary Fig. 11. Summary of various ligand-receptor interactions between cell clusters in pre-RCT (left panel) and post-RCT samples (right panel).**
