# Supplementary figures and images for: Single-cell RNA-sequencing reveals radiochemotherapy-induced innate immune activation and MHC-II upregulation in cervical cancer
Source: Signal Transduct Target Ther. 2023 Jan 30;8:44. doi: 10.1038/s41392-022-01264-9 (PMC9884664; doi:10.1038/s41392-022-01264-9)

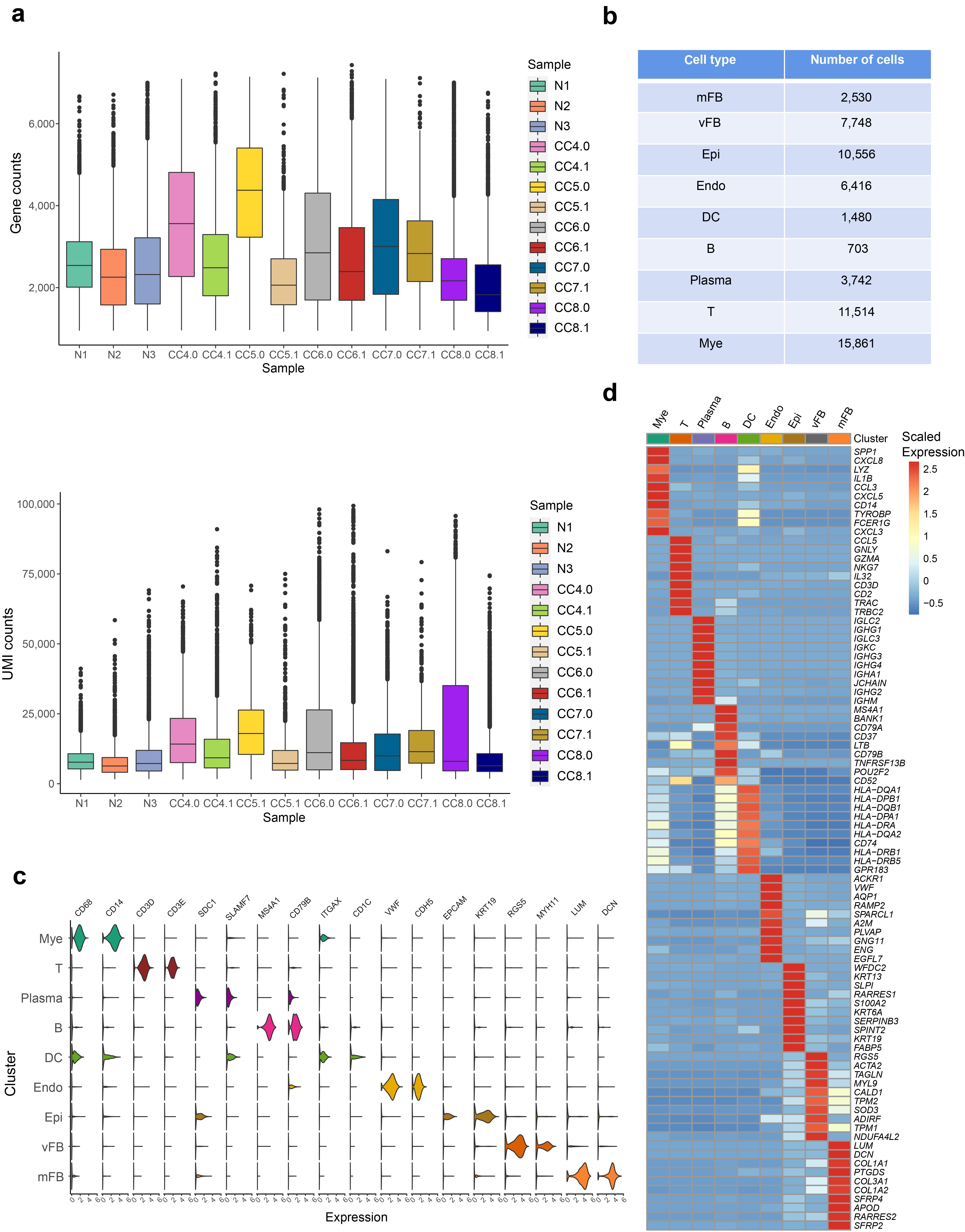

Supplement: Supplementary file 2 — Supplementary Fig. 1 [file 41392_2022_1264_MOESM2_ESM.jpg]

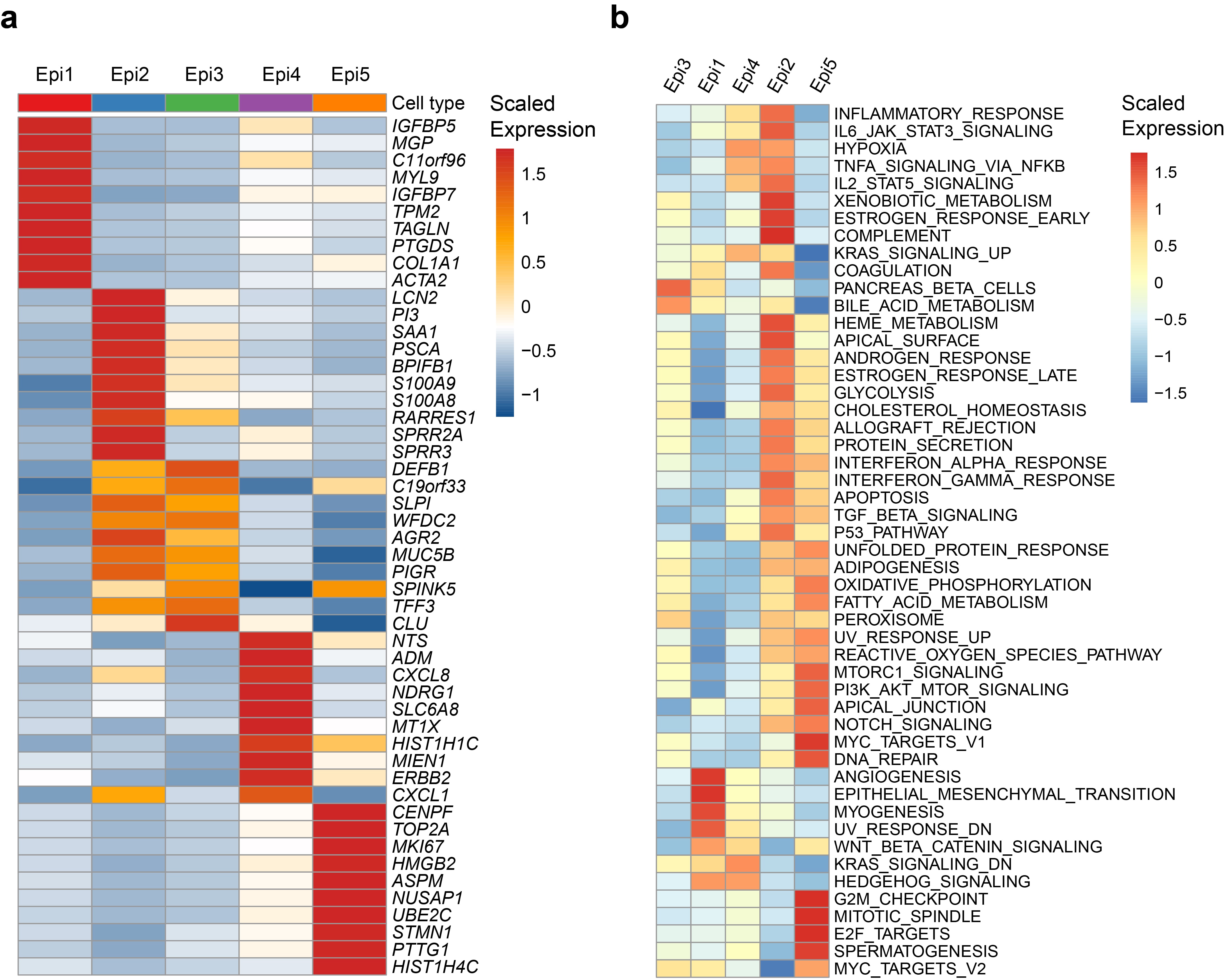

Supplement: Supplementary file 3 — Supplementary Fig. 2 [file 41392_2022_1264_MOESM3_ESM.jpg]

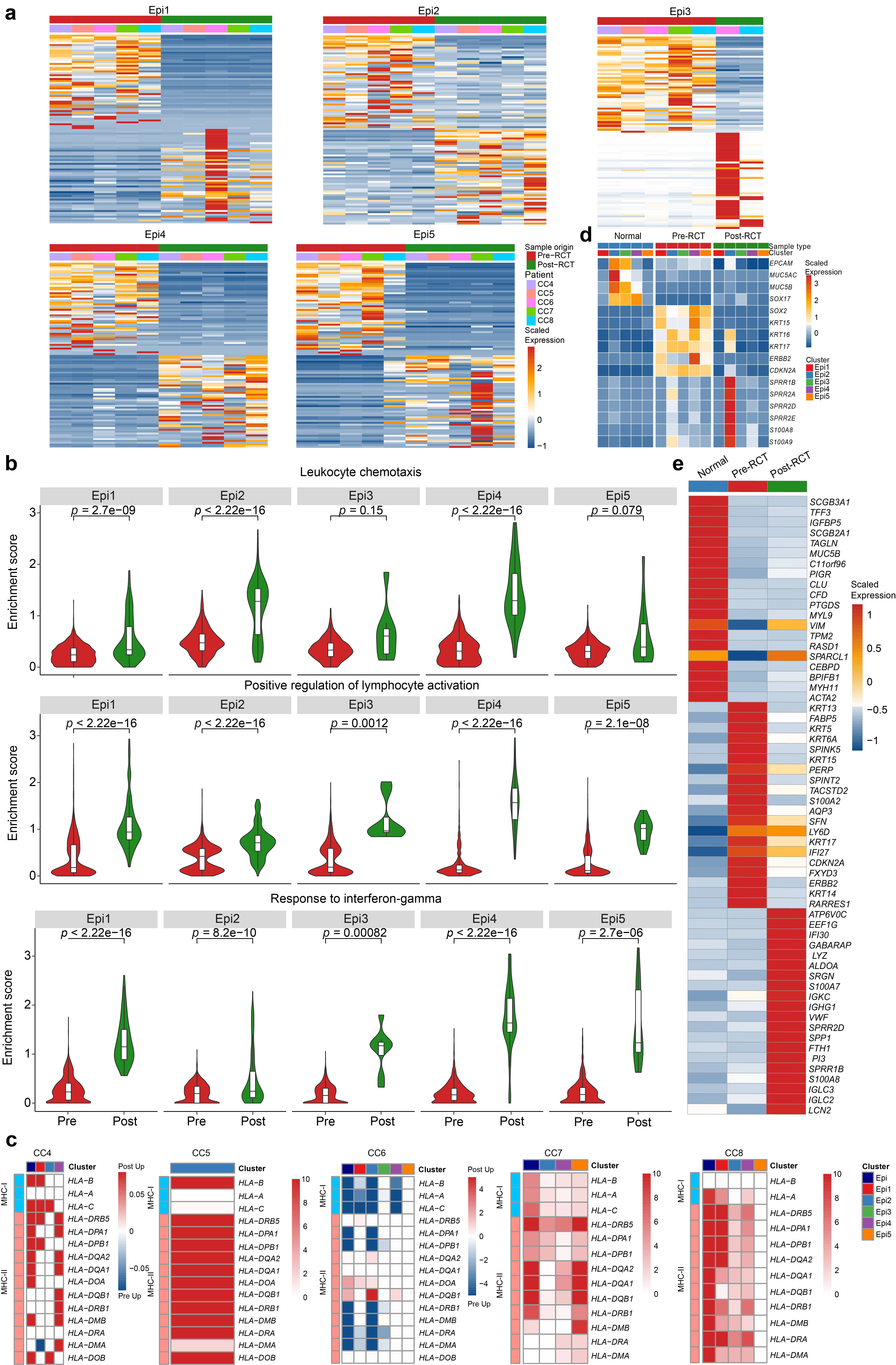

Supplement: Supplementary file 4 — Supplementary Fig. 3 [file 41392_2022_1264_MOESM4_ESM.jpg]

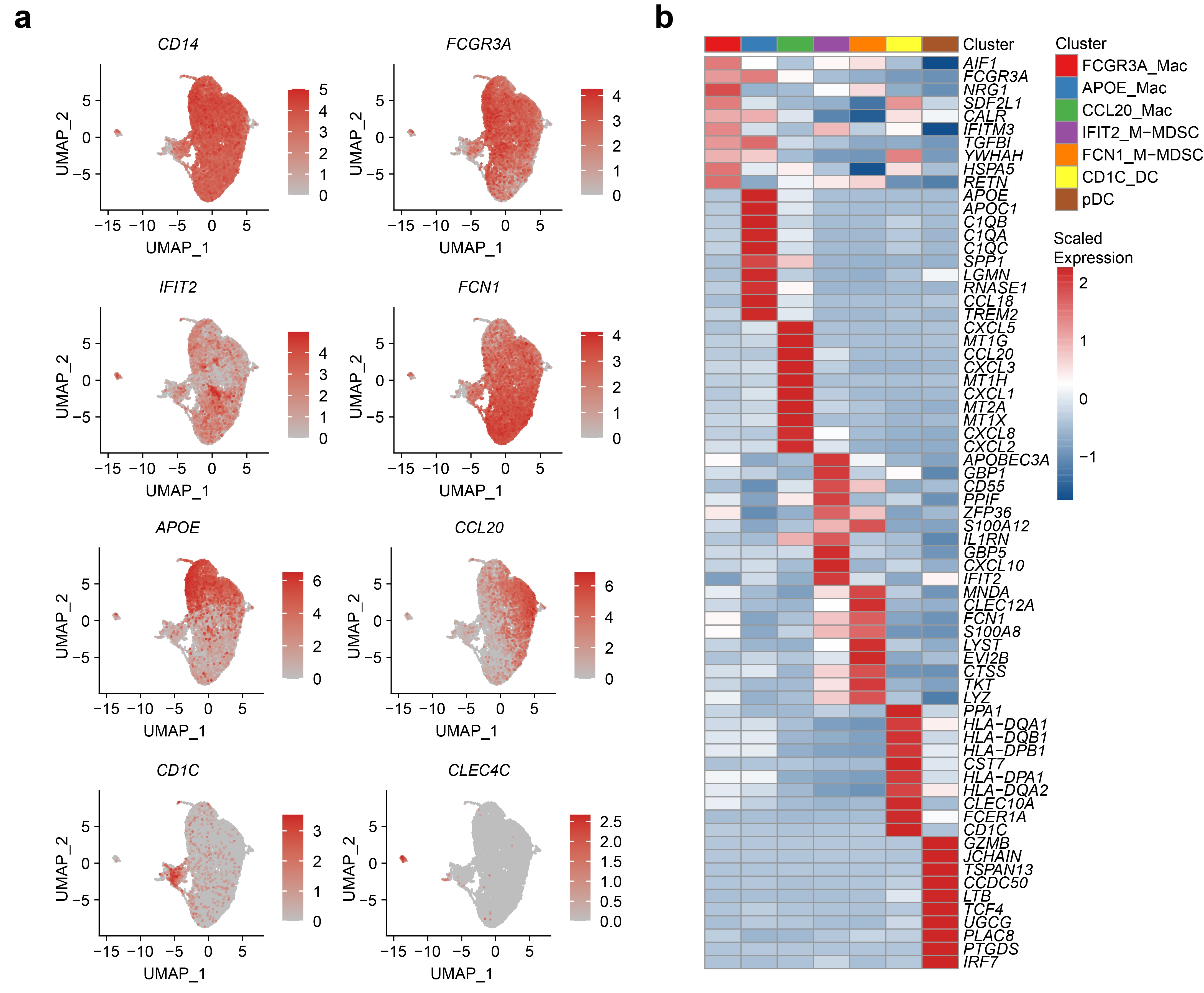

Supplement: Supplementary file 5 — Supplementary Fig. 4 [file 41392_2022_1264_MOESM5_ESM.jpg]

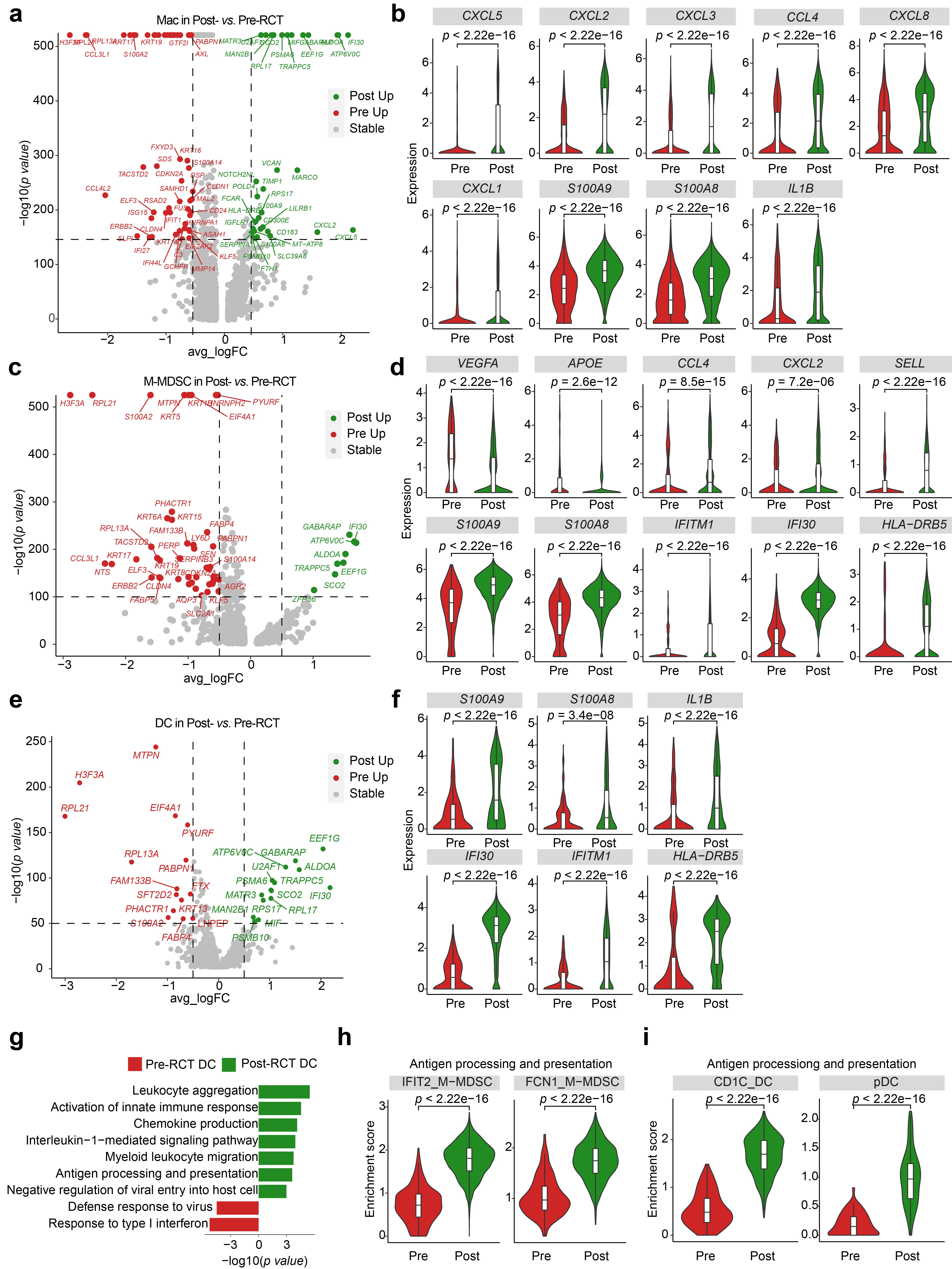

Supplement: Supplementary file 6 — Supplementary Fig. 5 [file 41392_2022_1264_MOESM6_ESM.jpg]

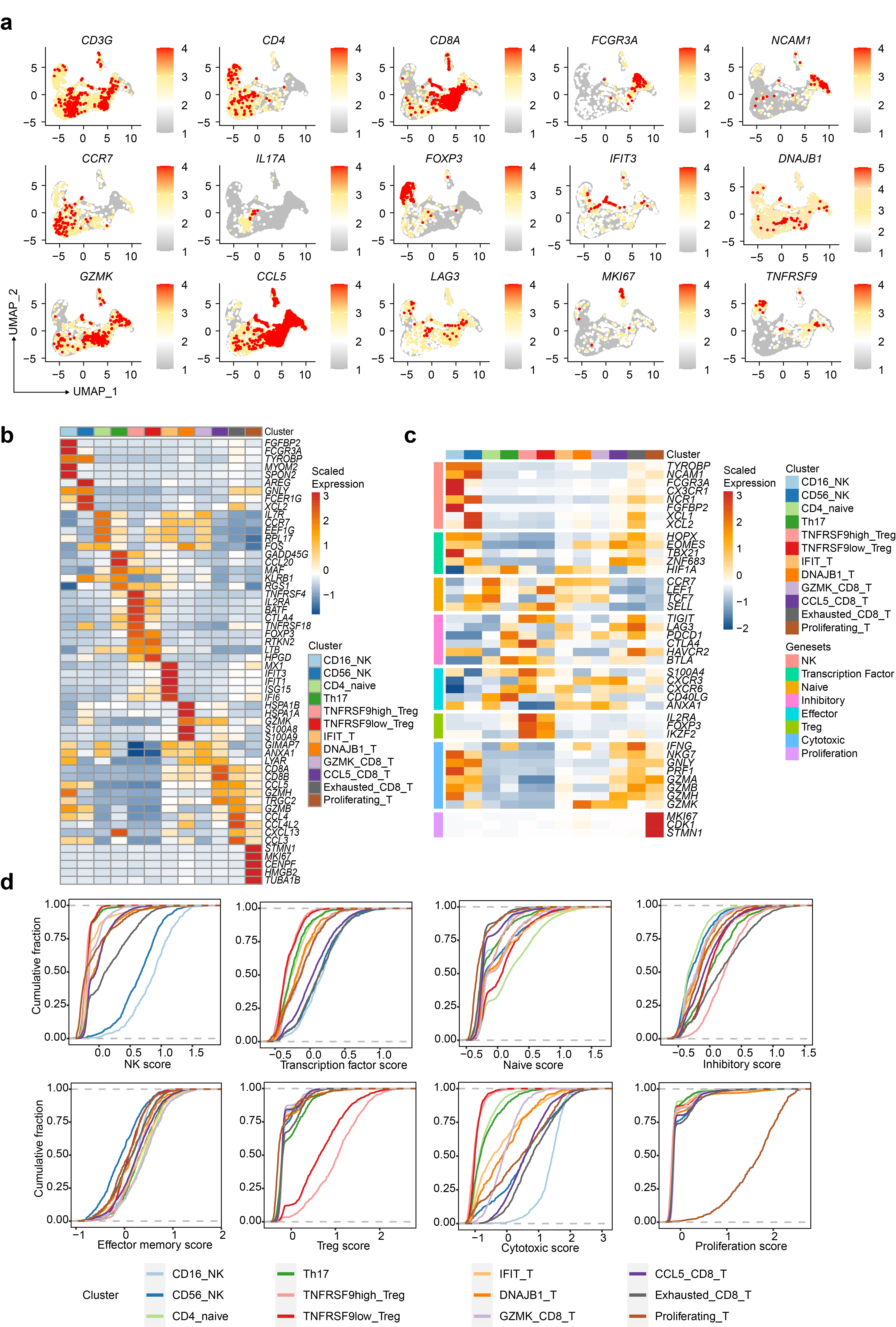

Supplement: Supplementary file 7 — Supplementary Fig. 6 [file 41392_2022_1264_MOESM7_ESM.jpg]

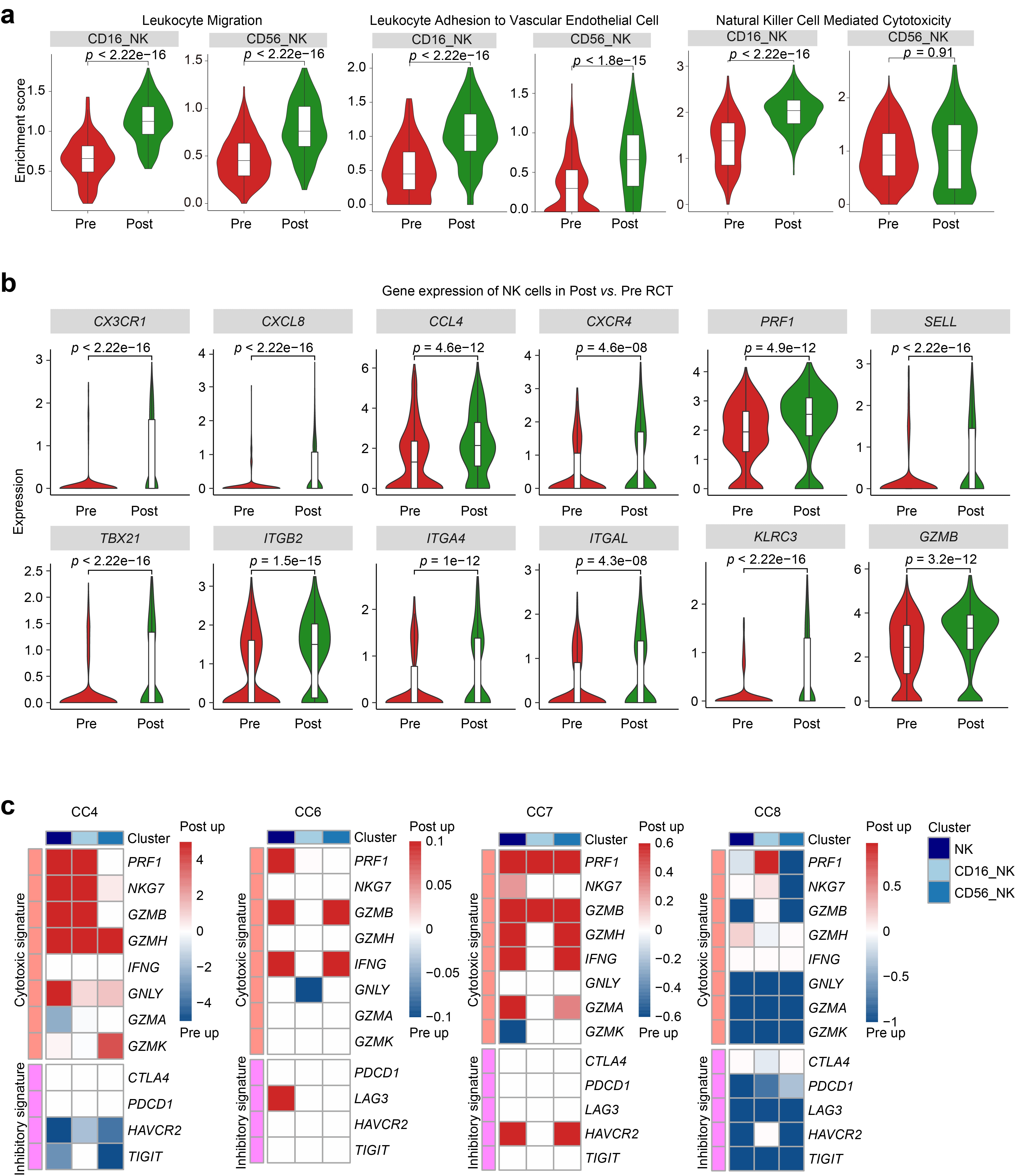

Supplement: Supplementary file 8 — Supplementary Fig. 7 [file 41392_2022_1264_MOESM8_ESM.jpg]

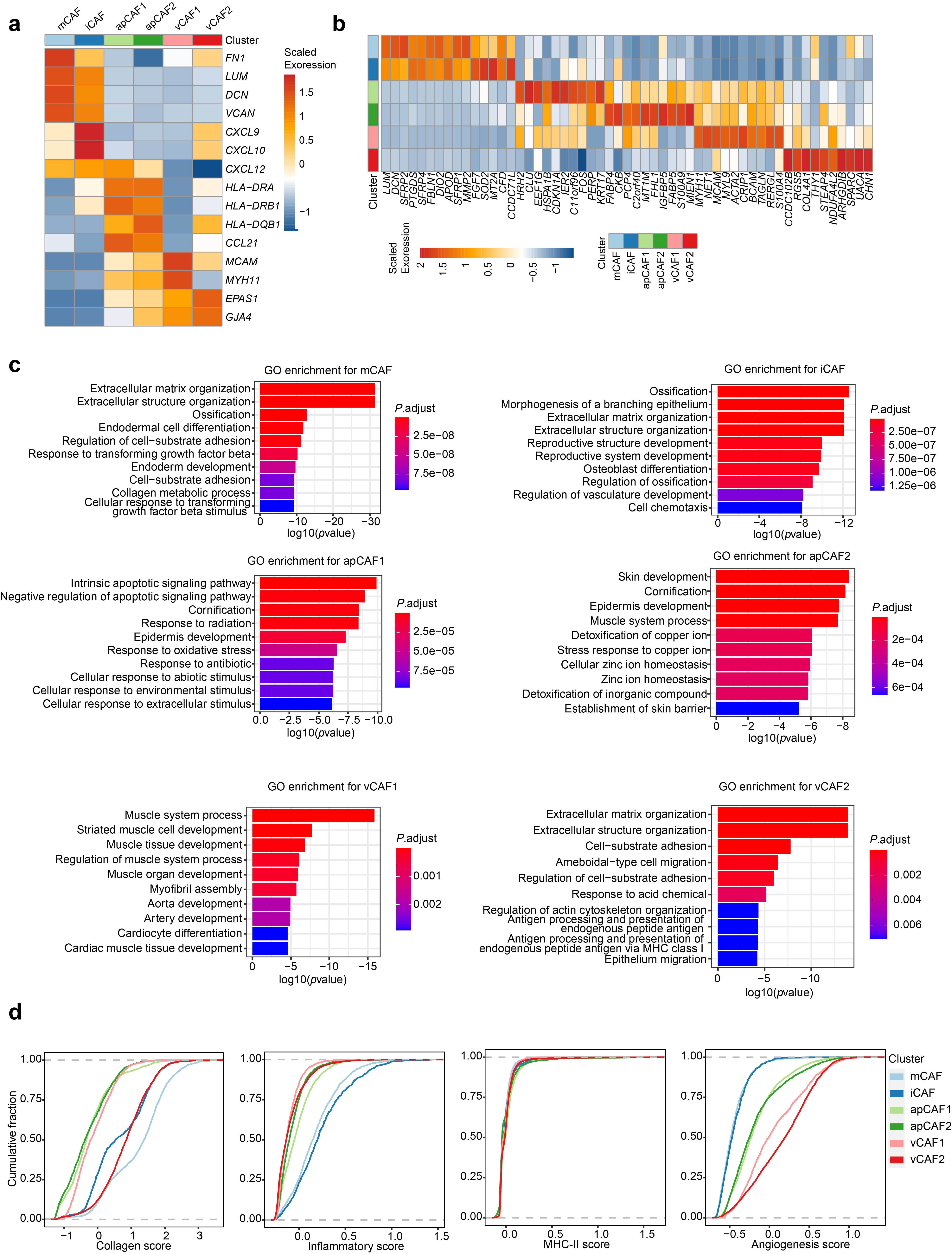

Supplement: Supplementary file 9 — Supplementary Fig. 8 [file 41392_2022_1264_MOESM9_ESM.jpg]

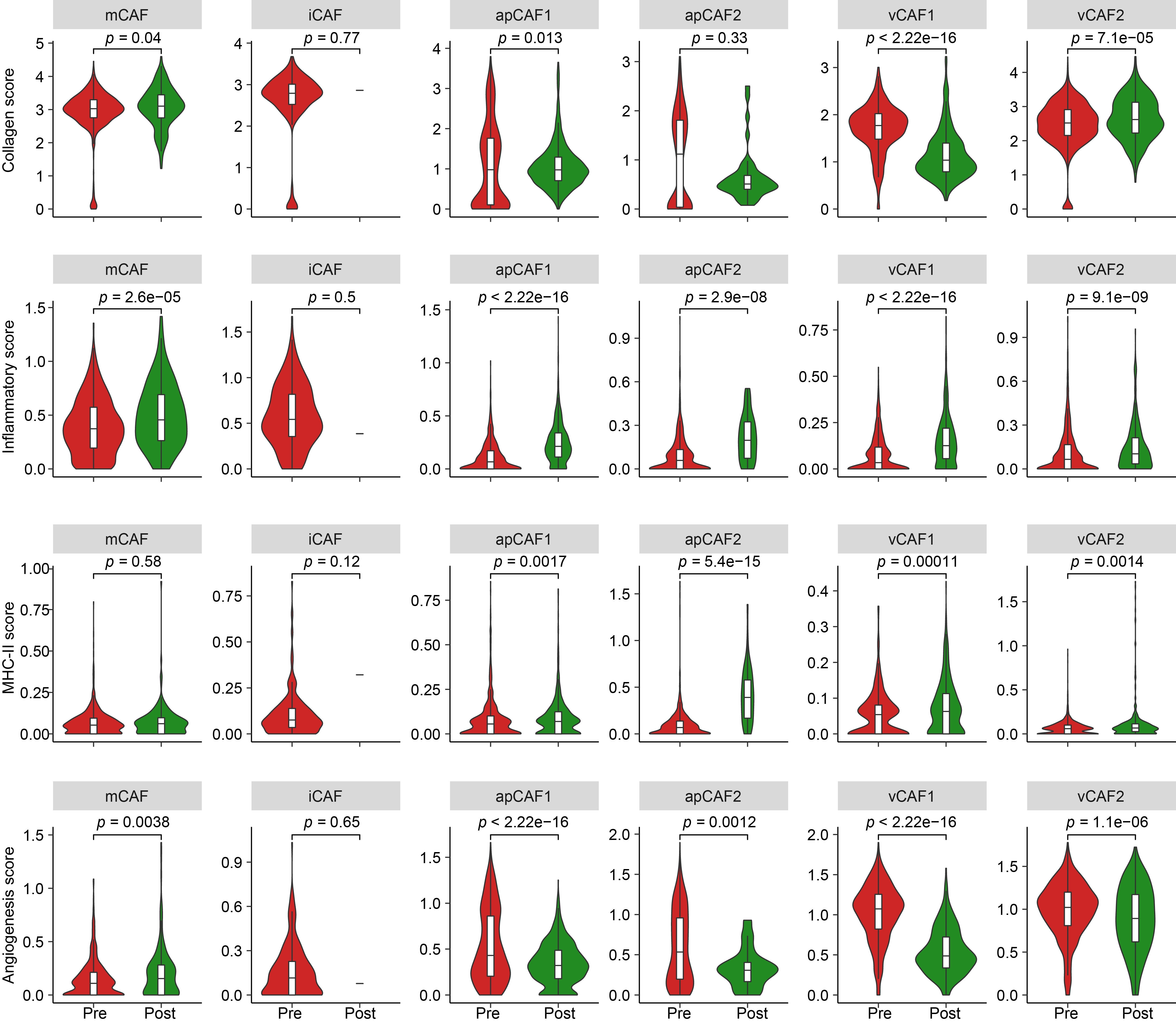

Supplement: Supplementary file 10 — Supplementary Fig. 9 [file 41392_2022_1264_MOESM10_ESM.jpg]

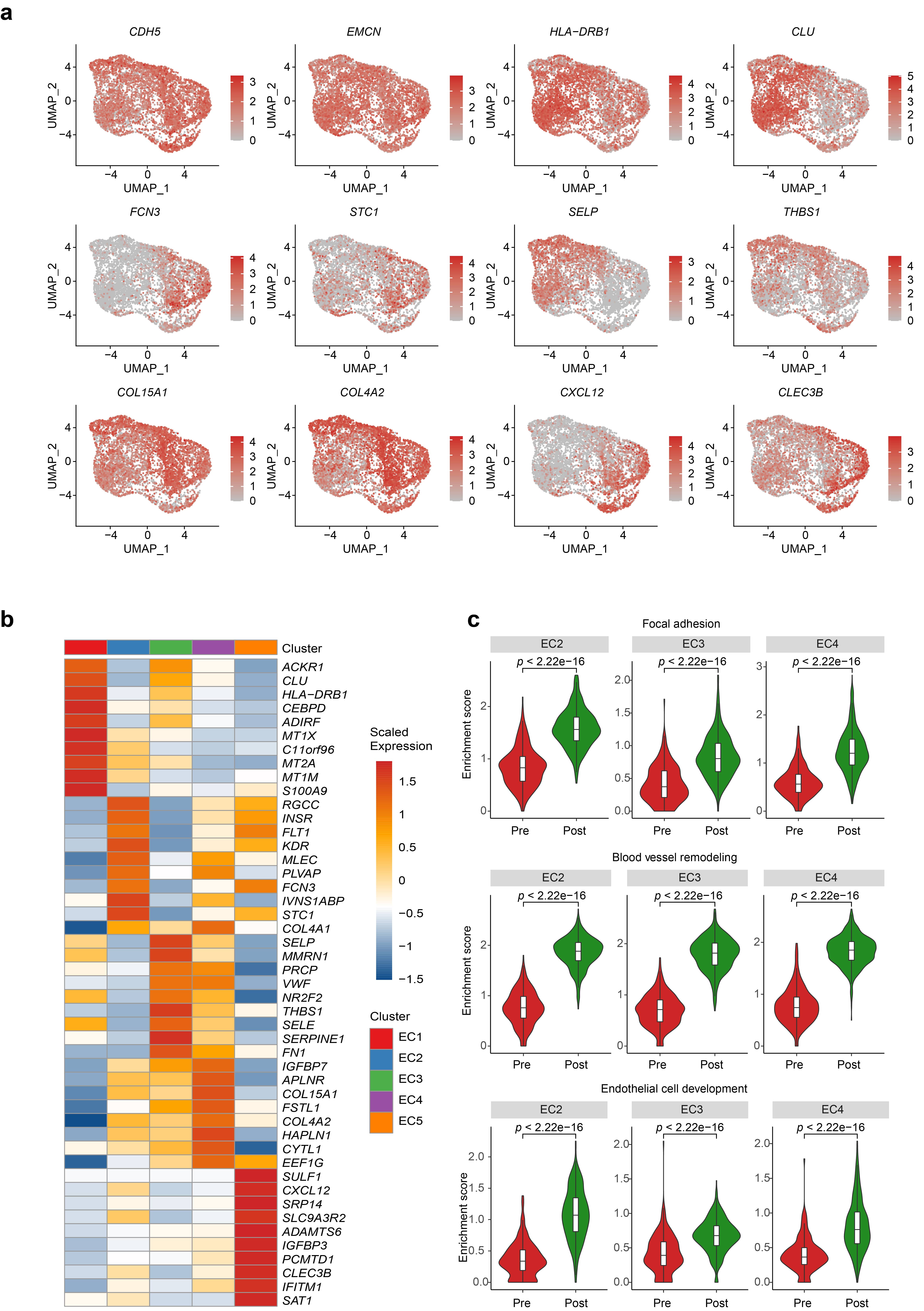

Supplement: Supplementary file 11 — Supplementary Fig. 10 [file 41392_2022_1264_MOESM11_ESM.jpg]

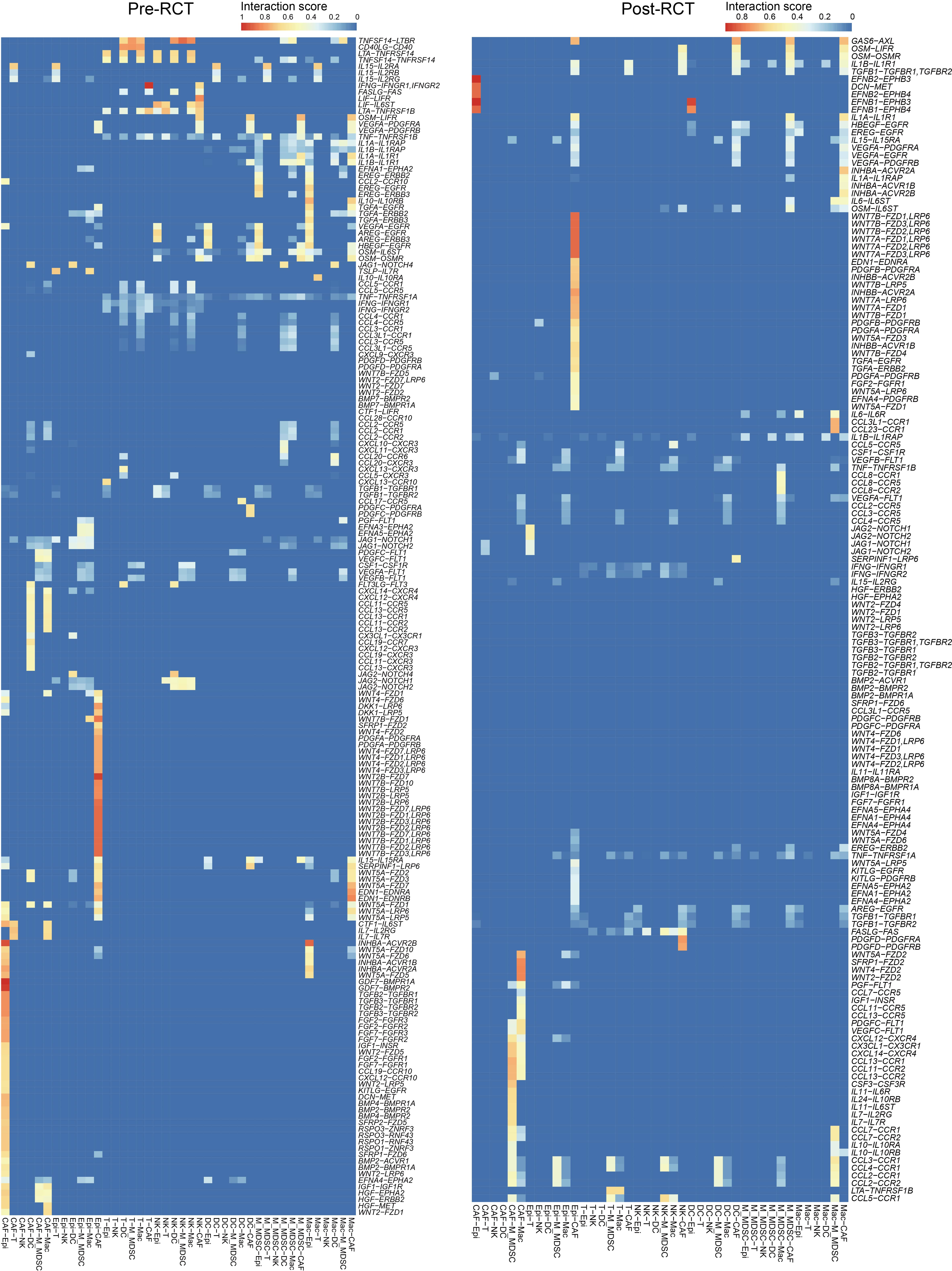

Supplement: Supplementary file 12 — Supplementary Fig. 11 [file 41392_2022_1264_MOESM12_ESM.jpg]
